# Electrochemical photonics: a pathway towards electrovariable optical metamaterials

**DOI:** 10.1515/nanoph-2023-0053

**Published:** 2023-04-27

**Authors:** Joshua B. Edel, Ye Ma, Alexei A. Kornyshev

**Affiliations:** Department of Chemistry, Faculty of Natural Sciences, Imperial College London, Molecular Sciences Research Hub, White City Campus, Wood Lane, W12 0BZ, UK; Department of Materials Science and Engineering, Ocean University of China, Qingdao, 266100, China

**Keywords:** controlled self-assembly, electrochemical cells, plasmonic nanoparticles, switchable mirror/window, variable-colour mirror

## Abstract

This review article focuses on the latest achievements in the creation of a class of electrotuneable optical metamaterials for switchable mirrors/windows, variable colour mirrors, optical filters, and SERS sensors, based on the voltage-controlled self-assembly of plasmonic nanoparticles at liquid/liquid or solid/liquid electrochemical interfaces. Practically, these experimental systems were navigated by physical theory, the role of which was pivotal in defining the optimal conditions for their operation, but which itself was advanced in feedback with experiments. Progress and problems in the realisation of the demonstrated effects for building the corresponding devices are discussed. To put the main topic of the review in a wider perspective, the article also discusses a few other types of electrovariable metamaterials, as well as some of those that are controlled by chemistry.

## 1 Introduction

Electrochemistry is a science that underpins the function of batteries, fuel cells, capacitors, solar cells, blue energy generation, electrolysis and photoelectrolysis for hydrogen production, water desalination devices, metal treatment, protection against corrosion, and electrocatalysis. Indeed, electrochemically based technologies are constantly being made more efficient, economically viable, and environmentally friendly. Their progress is visible. We see on our streets an ever-growing number of battery-powered modes of transportation including electric/hybrid cars and buses, electric bikes and scooters, along with the occasionally observed experimental fuel cell buses, vans and cars. We also heard of electrochemical-supercapacitor-driven trams (in China) recharging at each stop, construction cranes (in Estonia) recuperating energy by charging supercaps when laying down their platforms before loading, installations for the production of freshwater from sea brine (in Israel) or even from human urine (in space-crafts and the international space station). Of course, solar panels are also being extensively used in public and private households and institutions.

All of this became a reality due to the achievements of electrochemistry. This was brilliantly formulated in the historic lecture “Word on the value of electrochemistry” by A.N. Frumkin in 1975 at Mendeleev Congress in Alma Aata [1], who 57 years ago prophesied in fine detail the future of an electrochemical-technology reliant world that we see today. Also recalling the important potential of electrochemistry for the food industry: electrochemical synthesis of ammonia for fertilizers avoiding the production of CO_2_ as a side product, and of course the mentioned desalination of water that potentially opens unlimited resources of extracting fresh water from the ocean, as well as unlimited resources of “blue energy”. One should admit that electrochemistry can be regarded as the “cleanest” chemical science. This point was neither missed in Frumkin’s lecture, who put up an unforgettable metaphor in the face of oil and gas chemistry dominated academics and industry: “Aphrodite came out of sea froth – a concentrated electrolyte solution, but not from oil”. This was particularly visionary especially when people did not seriously think about the environment and did not know much about climate change.

In the fundamental electrochemistry underpinning all those applications, the key factor is the potential distribution across electrode/electrolyte interfaces. In particular, the potential drop between the electrode and the position where reactants settle near it is the main driving force in electrochemical kinetics and electrocatalysis. It has taken approximately 170 years and great minds to understand and rationalize this [2], however, not all the details of this potential distribution are clear even today [3, 4]. Understanding its details or not, we all know how important it is. In the 1980s, when physicists actively entered electrochemical science [5] triggered by the discovery of electrochemical Surface Enhanced Raman Scattering (SERS) [6], they teasingly called electrochemistry a “*surface science with a joystick*”. By the “stick”, they meant potentiostat that controls the potential drop across the electrochemical interface and thereby dramatically affects the processes that take place at the interface, being able to turn them on and off. That option was not available in vacuum surface science at that time, and even nowadays only partially available in solid-state nano-junctions.

Physicists realised the power of that “joystick”. Indeed, with just a 1 V voltage drop concentrated at the electrode/electrolyte interface within the electrical double layer extending to just 1 nm for a typical electrolyte concentration (0.1 mol/L), one gets a highly localised electric field of some 10^7^ V/cm. This field can be easily controlled by variation of applied voltage within the same ±1 V. Typically within this or slightly smaller range, no electrochemical reactions take place in response to charging of the electrode, but only the rearrangement of ions (enrichment of the double layer with ions of opposing charge – the *counter-ions*, and depletion with the ions of the same sign– the *co-ions*), which is used in storing energy in electrochemical capacitors. This voltage range is called the *capacitance window.* Going beyond that window with full voltage control, still within a few volts of variation of the electrode potential was key for the electrocatalysis of reactions at the electrochemical interface. But is driving electrochemical reactions or storing energy by polarizing the electrochemical interface as well as other mentioned applications, all that such systems could do? Over the last 40 years, it was understood that it could do more. Not unrelated to electrocatalysis is the science and technology of electrochemical sensors. One example is the invention of the blood glucose sensor which transformed the quality of life for diabetics worldwide by allowing people to monitor and record their blood glucose levels [7]. For reaction-free applications, based on the rearrangement of ions under applied voltage, one can also mention the effect of shape change caused by recharging the electrical double layers between the anode and cathode separated by an ion-conducting membrane matrix, i.e. the *electroactuation* [8] which can be used in micro-robotics. The reverse process – a mechanically forced change of the contact between electrode and electrolyte under constant voltage, can change the electrical capacitance of the system and generate electrical currents in the external circuit. Such *reverse actuation* can be used to generate electricity for portable electronic devices from otherwise wasted mechanical motion, e.g. from walking [9–11]. Potential variation across the double layer can be used as a gate voltage, which controls the performance of *nano*- or *single-molecule diodes* [12]. These are simply a few examples. In the context of this review, the most interesting option is that it can provide controlled self-assembly of charged nanosized particles at the electrochemical interface. But what’s in it for optical applications?

The definition of optical metamaterials was well formulated in Zheludev’s manifesto note [13]. “Metamaterials are artificial media structured on a scale smaller than the wavelength of external stimuli. Conventional materials derive an origin for their electromagnetic characteristics in the properties of atoms and molecules – metamaterials enable us to design our own ‘atoms’ and thus access new ground-breaking functionalities such as invisibility and imaging with unlimited resolution”. Currently, many of such materials are based on sophisticated, cutting-edge nanotechnology, their architecture is composed of nanosized building blocks, constructed, usually “once and forever”. Therefore, in the same note, Zheludev wrote “The next stage of the photonic technological revolution will be in the development of active, controllable … metamaterials”. He reiterated this in his opening speech at the Meta 2014 conference in Singapore, addressing the participants with a provocative statement “The time of metamaterials is over. It is the time of…tuneable metamaterials”. Since that time, and to some extent before then, there was a steady flow of works in this direction, many of which were based on the optical response to micro-mechanical deformation of metamaterials, but some used more complicated electrodynamic effects [14–43].

Electrochemistry can contribute to the tunability of metamaterials. The idea to use its potential is directly related to *self-assembly*. Indeed, instead of constructing optical metamaterials, which is a great task in itself, one may create conditions to enable their self-assembly? Controlling such conditions would help to tune their properties. If the self-assembly is reversible, such control can turn the properties of the material on and off. Applying external fields – electrical or magnetic may help to change their properties in real-time. As we will describe in this review, at least in part, such ideas have been realised in their simplest form: (i) spontaneous self-assembly of arrays of *charged* plasmonic **n**ano**p**articles (NPs) at liquid/liquid interfaces and (ii) the voltage-controlled self-assembly of NPs at *electrochemical* liquid/liquid and solid/liquid interfaces. As described above, at electrochemical interfaces the ability to control the electric field in the double layer helps to achieve the voltage control of the structure of such arrays, making their optical response electrovariable.

The physics of it is simple. In brief, NPs are deliberately made charged to repel each other, in order not to fuse in the bulk of the liquid due to attractive Van der Waals forces. This is typically achieved by functionalizing NPs with ligands, the terminal groups of which dissociate and thus get charged – the value of the charge being controlled by the ligand and solution pH. The same charge acts as a “good cop” and “bad cop”: it provides the needed response to the electrical field in the double layer, but it also tends to prevent nanoparticles from settling too close to each other at the interface. Noteworthy, each individual, charged NP may or may not spontaneously adsorb at the interface, but the electric field at the interface can, respectively, affect or create the potential well that would keep the NP there. Changing the polarity of the electric field can equally destroy that well, pushing NPs away from the interface. But NPs do not adsorb at the interface alone. Charged in the same way, they repel each other in the adsorbed layer, making the wells keeping each NP at the interface shallower or none. But, again, varying the electric field at the interface, can make those wells deeper, competing with the Debye-screened Coulombic repulsion between the nanoparticles, or shallower. Making the well shallower or completely eliminating it, with the consistent electrostatic repulsion between the NPs would allow the formation of very sparse assemblies or even the complete release of nanoparticles from the interface to the bulk of the solution. But, as in life, both “good” and “bad” cops are needed for the case, both factors are essential for the control of the NP arrays. It is the electrostatic repulsion between NPs that warrants the homogeneity of the array, its monolayer character, and the variability of the array density under the response of the charge of each nanoparticle to the external field.

This article will overview the progress towards electrotuneable mirror-windows, variable colour mirrors, optical filters, and antenna structures for Raman sensors. We briefly, and only in qualitative terms, will characterise the theory underpinning these systems, referring the readers to the corresponding original papers, and discussing the first, proof-of-the-principle experiments. It is already a long time passed since the first reviews on this topic [44, 45], and the goal of this article is to update the readers on the latest achievements and the key obstacles that one needs to and could overcome on this front.

Before we start, a comment on the title of this article:

The adjective “electrochemical” does not mean that manipulation with self-assembled structures involves electrochemical reactions. Most of the methods described below deliberately avoid the latter, operating within the mentioned “electrochemical” or “capacitance” window of applied voltages, when on solid electrode/electrolyte interfaces no reactions involving solvent molecules or ions yet turn on. Whereas at liquid|liquid interfaces, no ion transport across the interfaces is triggered by the applied voltage. This is because such processes will disturb the system and distract from the main goal of rearranging the metastructures, in the long term leading to the degradation of the corresponding devices. Avoiding such processes used to be called: “working in the *double layer regime*”. In such a “physical regime” of electrochemistry, the right “*chemistry*” is still very important. It is related to the types of electrolytes and solvents, functionalization of NPs by ligands, and solution pH – all crucial for the precise control of the electric field at the interface under the applied voltage.

## 2 Two dreams

**Figure 1:**
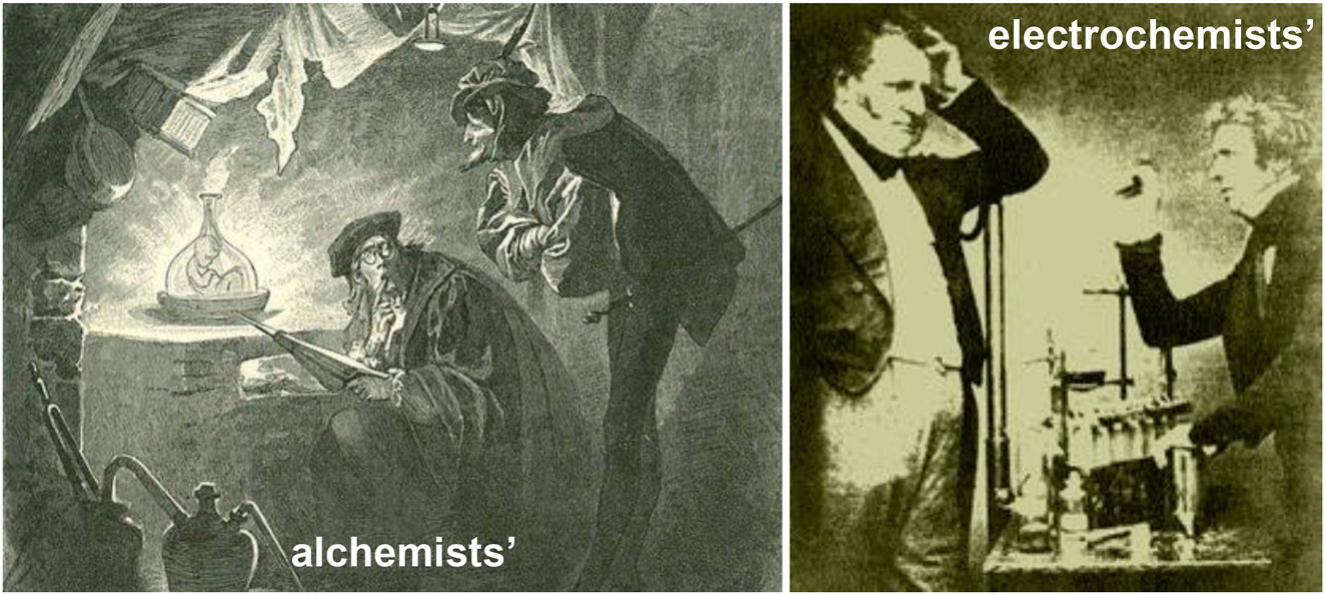
Two dreams: the dream of a medieval alchemist and the dream of an electrochemist.

Electrochemistry is in the unique position to offer new scenarios for the *tunability of metamaterials* along with two alternative scenarios*.* Their nature goes back to old times (Figure 1).

*A dream of a medieval alchemist* was to find a magic composition of a solution under which a homunculus will self-assemble in a test tube or that iron (or at least silver!) can be converted into gold*.* Whereas the former search is still on the way using so-called synthetic biology (e.g. construction of artificial cells), the former process is unlikely ever to be achieved, seemingly forbidden by the laws of nature. But the mere idea of self-assembly, pioneered in those old days, is widely used in modern soft matter physics, polymer science, colloid science, and supramolecular chemistry. It had neither escaped the attention of electrochemists [46]. We will describe below how it can be used in photonics.

*A dream of an electrochemist*, since Faraday times, has been to apply and vary the voltage in an electrochemical cell, and whatever self-assembles or disassembles at the electrochemical interface will depend on the electrode potential. Paraphrasing Feodor Dostoyevsky, who said that “Beauty will save the World”, a maverick electrochemist would say the same about their potentiostat. The realization of this second dream is a continuous process.

We will consider below the use of both approaches to control the plasmonic effects [47].

## 3 Plasmonics and electrochemistry

*Plasmonics* is a branch of solid-state physics and optics exploring different effects of collective electron plasma excitations (*plasmons*) in the optical response of interfaces and interfacial films and exploiting them in various optical, sensing, and catalytic processes [48]. Plasmonics took off in the 1960s–1970s, with the pioneering works of Otto [49] and Kretschmar [50, 51] who formulated and demonstrated the principles of techniques for the characterisation of surface plasmons and experimental measurements of the spectra of these collective excitations, whereas the concepts of bulk plasmons and detection of surface plasmons in thin films were already proposed in 1950s [52, 53].

Plasmonics and electrochemistry got acquainted with each other more than 40 years ago, upon the discovery of Surface-Enhanced Raman Scattering (SERS). The famous paper of the Southampton electrochemistry team – Fleischman, Hendra, and McQuillan [54] was the first to report a remarkable observation of a “giant” enhancement of Raman signal from pyridine molecules adsorbed on a rough silver electrode. However, that outstanding paper had not unravelled the nature of this effect. On the West side of the Atlantic, they use to say that Fleischman et al. *observed* the effect, whereas Van Duyne [55] at the Chemistry Department of North Western University, with his systematic studies, has laid the foundation of understanding what stands behind it/and proved what it is not. They tend to attribute the discovery of SERS to him and acknowledge those who contributed to its scientific understanding (see pioneering theoretical works of Schatz, Moskowitz, and others, as reviewed in [56–58]). Indeed, it had slowly become clear that the giant enhancement of the Raman signal from molecules adsorbed on rough surfaces is in the first place related to plasmon resonance due to a possibility of direct excitation of surface plasmons by light incident onto a *rough* metal surface. First estimates, confirmed later with the development of computational methods for solving Maxwell equations, made it clear that local values of the electric component of electromagnetic radiation, *E*, with frequencies close to surface plasmon ones, maybe resonance-enhanced a couple of orders of magnitude near the tips and in crevices of the rough metal surface. Since the intensity of the Raman signal from an analyte molecule scales roughly as *E* ^4^, one can easily end up with 10^8^ times enhancement of the signal. Other factors responsible for the enhancement are associated with the so-called “chemical” or charge transfer enhancement, as rationalised by Albrecht and Creighton [59], and later by Otto [60]. Plasmon-resonance mediated enhancement remains central for SERS. In the 1980s interest in plasmons in electrochemistry beyond SERS, boosted research in electrochemical electromodulation spectroscopy [61–65].

The relatively recent boom in nanotechnology and nanoscience, gave rise to the second birth of plasmonics, more generally photonics which includes plasmonics as a particular subject. Indeed, conventional materials derive their optical properties from atoms and molecules. Nano- and micro-technology could create structures and architectures from larger blocks than simple atoms, e.g. nanoparticles or other components of nanostructures, thus designing larger scale “atoms” to access new functionalities that capitalize on plasma excitations in these complicated structures or more complex effects [66]. Thus the science of metamaterials was born, and extended nowadays to quantum metamaterials, nonlinear and amplifying metamaterials, materials for transformation optics, invisible cloaks, designer dispersions for slowing light, chiral metamaterials, sensor metamaterials (e.g. based on SERS), microwave frequency selective materials, complex optical filters, light trappers, etc… [13]. All of these are the key components of what nowadays is called *photonics* [67, 68], the term widely used to embrace the directions of modern optics that use metamaterials or tricky electrodynamic phenomena to produce novel optical effects.

Electrochemistry very often rapidly followed discoveries in physics, and there are many examples of it in the history of science. Take for instance building the *in situ* Scanning Tunneling Microscope (STM) with atomic resolution which could picture atomistic structure of the electrode/electrolyte interface [69, 70], just two years after Binning and Rohrer reported their vacuum STM results [71, 72], or fast implementation of discovered carbon nanotubes or graphene for building electrodes for electrochemical supercapacitors [73]. Electrochemistry is moving towards further exploiting the achievements of modern plasmonics; for instance, a new field of *reactive plasmonics* was recently born [74–76], and one of its goals is to explore and exploit the effects of giant local enhancement of the electrical component of electromagnetic radiation in photoelectrocatalysis [77, 78] or at least optically identify intermediates of such reactions [79, 80]. Whatever may come out of the marriage of plasmonics and electrochemistry can be achieved by using nano-engineered electrodes [81–83] or playing with self-assembling nanostructures, which is the focus of this review.

## 4 Controlled self-assembly of nanostructures at liquid|liquid interfaces

The simplest of such self-assembling nanostructures were arrays of metallic NPs at the **i**n**t**erfaces of **i**mmiscible **e**lectrolytic **s**olutions (**ITIES**), otherwise called electrochemical **l**iquid/**l**iquid **i**nterfaces (LLI). The road to these works was opened by pioneering papers of the groups of Schiffrin et al. [84–88] who have studied the formation of such layers at LLI [89, 90], including the first results of their optical and/or electrical characterisation. Indeed, properly functionalised NPs usually dispersed in the liquid phase tend to spontaneously adsorb onto the LLI to block the unfavourable contact between water and oil. Voltage control over the electrosorption of NPs was demonstrated initially for 1.5 nm-sized NPs. Those were, however, too small to see any noticeable contribution to the modification of the linear optical signals. The voltage effect has been first demonstrated through cyclic voltammetry and capacitance measurements [59]. Second-harmonic generation detecting the presence of NPs at the LLI was first demonstrated in Ref. [58], but again for relatively small NPs, 6 nm in diameter.

In 2010, Flatte, Kornyshev, and Urbakh proposed and explored theoretically an idea on how to influence optical properties of ITIES through voltage-controlled assembly/disassembly of NP arrays, by tuning both the concentration of the electrolytes in the two solutions and the potential drop across the interface [47]. With the preliminary character of the rough estimates presented in that *Feature Article* and some minor inaccuracies there, this theoretical paper launched a new direction of research: *electrotuneable electrochemical plasmonics*.

Indeed, dense quasi-2d NP arrays of plasmonic NPs (gold, silver, titanium nitride, core–shell composite NPs, etc.) adsorbed at the interface of two optically transparent media reflect light in a broad visible range with a maximum reflection centred about the frequency of the localised plasmon mode in NPs. That frequency depends on the size and the dielectric properties of the material. For 20 nm diameter gold NPs (AuNPs), it is close to the wavelength of 560–580 nm. The overall reflection signal also depends on the material and size of NPs: the intensity of the reflection is weaker, the smaller the size and the stronger the light absorption in the NP material. Too large sizes of NPs, approaching the wavelength of light, would neither be good for reflection, as this would give rise to diffuse scattering of light. The size between 16 and 40 nm in diameter was found to be most suitable for the study of enhanced reflection. Apart from the size, another decisive parameter is the average distance between NPs in the array, i.e. the array density. Increasing the array density affects the reflection spectra in two ways: (i) the reflection across the whole spectrum increases with density; (ii) the maximum wavelength shifts to the red.

**Figure 2:**
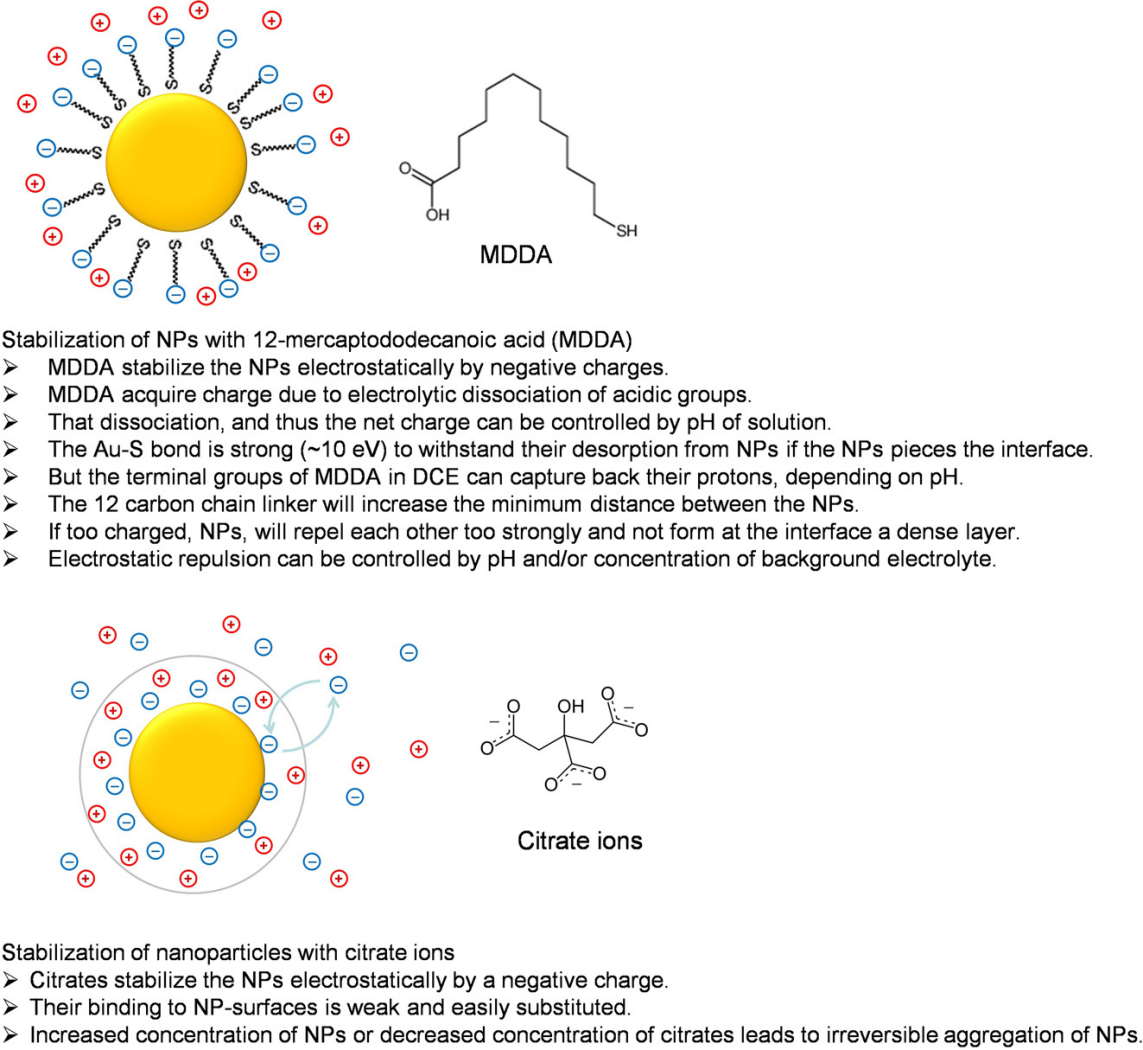
Two possible ways of functionalization of nanoparticles, making them “charged”.

The following strategy and manipulation schemes were suggested, which were later approved by a series of systematic experimental studies:

– NPs are functionalized with ligands, such as mercaptanoic acid or citrates, as sketched in Figure 2. Both these substances dissociate in water, retaining negative charges on ligands associating with NPs. Some other substances may be used resulting in positive charges of ligands. The sign of the charge is not principle, but rather its value, as long as that sign is the same for all NPs. The charge thus associated with NPs will protect them against agglomeration that may be caused by Van der Waals forces, which is a standard measure for the stabilization of colloids. A 20 nm in diameter NPs functionalized at neutral pH by mercapatanoic ligands can bear approximately 900 elementary charges.
– The electrostatic repulsion of charged NPs can be controlled through the variation of electrolyte concentration that affects Debye screening and through the degree of ionization of the ligands, which can be altered by adjusting the pH. A higher electrolyte concentration will screen stronger the electrostatic repulsion in the solution. More acidic pH will reduce the degree of dissociation of acidic terminal groups of functionalised ligands and decrease the overall charge associated with NPs. Both factors will reduce the repulsion of NPs in the array adsorbed at the interface and would favour denser arrays. But obviously, this cannot be overdone – too short screening lengths in the solution and a too small charge on the NPs would lead to their agglomeration in the bulk.
– The control of inter-NP spacing through electrolyte concentration and pH is, thus limited. Although each NP would wish to settle at the LLI, the system should be designed in such a way that NPs still repel each other strongly enough not to fuse with each other in the bulk. Therefore, they will tend to keep some “social distance” between each other when adsorbed at the interface. Thus, even in the absence of any applied bias voltage the strategy is to reduce repulsion as much as possible, but not that much that they would start agglomerating in the bulk including at the LLI. Theory must try to estimate the limiting values for electrolyte concentration and pH, but those are best defined for each system experimentally, and that has been repeatedly done. Indeed, the agglomeration of NPs in the bulk can easily be detected through the characteristic features in the light extinction spectra of the solution bulk. All in all, each such system will have some minimal distance for interparticle separation in an adsorbed array. Since nanoparticles were designed to repel each other, for spherical NPs that array will be, on average, hexagonal.
– If we want NPs to form even denser arrays, a natural idea would be to apply a voltage across the ITIES to push the charge NPs to the interface. If the ligands are negatively charged, this will take place when polarizing the aqueous phase, in which the NPs are dissolved, negatively. When positively charged ligands are used, this should be the other way around. Polarising the cell correspondingly will increase the driving force for NPs to settle at the interface. The capillary well and solvation do not let them go into the oil phase completely, so they settle at the ITIES, piercing it, although when being pushed by an electric field they can be somewhat shifted to the oil side of the interface. This would make the potential well for each NP deeper, and thereby increase their ‘desire to settle at the interface’ so that NPs will tolerate shorter social distancing between each other.

These ideas were touched upon in the 2010 paper, however, all the quantitative details were made clear only after a series of follow-up theoretical works and experimental observations.

The theoretical work [91] focused first on the development of a more accurate theory to determine the optical signals obtained from arrays of spherical NP settling at optically transparent interfaces such as an LLI, testing it against full wave simulations. The theory itself had an “effective medium” character: it represented an array of NPs as an effective film, dielectric properties of which are determined by the dielectric properties of NPs, interactions of induced localised plasmon oscillation modes (for simplicity considered as dipolar ones) of each NP with those of all others. In addition, with the interaction of dipolar modes between NPs, interaction with their images across the interface was also taken into account in the calculation of the effective dielectric response of that effective film. As a result, the effective dielectric tensor of the film representing NP array at the interface appears to be anisotropic, with distinct normal and lateral components of the dielectric tensor, and it depends on the size and material of NP, the inter-NP separation, and position of NPs relative to the interface. Note that image interactions are less important for transparent interfaces, but they proved to be much more important when the same kind of theory was later extended for the NP arrays at the metal/electrolyte interface [92]. With an accurate approach for the description of the dipolar image forces and better approximations of plasmon resonances in individual NPs, the theory was brought to excellent agreement with full wave simulations [91, 92], as well as, later, experiments. The systematic realisation of these principles and tests of the theory started in 2015 at Imperial College London.

That period of work was preceded by a series of earlier works, performed before achieving electrovariability experimentally, and reviewed in Ref. [45]. It started with experimental investigation of the “chemical” factors influencing the adsorption of NPs onto an LLI including electrolyte concentration and the aqueous solution pH [93] followed by computational analysis of the optical properties of NPs at the interface [94], culminated in Ref. [95], which has demonstrated unprecedented levels of sensitivity for the detection of Raman signals from a series of analyte molecules adsorbed at the LLI in between 40 nm size AuNPs. Control of the density of the adsorbed NP-array was only via variation of electrolyte concentration and pH. But it has shown, how “hot” can be the spots of enhancement of electromagnetic radiation (near and especially in between the NPs), via probing the vibrational modes of the analyte molecules through their “Raman fingerprints”. Although that work did not involve an electrochemical setup, and thus no voltage could have been applied to stabilize and densify the adsorbed NP arrays, it was unambiguous evidence of the formation of relatively dense NP arrays at an LLI.

This was followed by a seminal paper published by the group led by Mark Schlossman at the University of Illinois in Chicago. They have performed fine investigations of the assembly of small (2 nm) NPs functionalized by positively charged ligands [96]. Their localization at the water/DCE ITIES was studied by Grazing Incidence Small Angle X-ray Scattering (GISAXS), controlled by the voltage applied across the interface. From Bragg diffraction peaks, they could see a hexagonal arrangement of NPs at the interface, with the decrease of the lattice constant, when NPs get pushed towards the oil by an electric field at the interface. Through X-ray reflectivity, they could also assess how deep the NPs can be pushed towards oil when polarizing water positively with respect to oil. The molecular dynamic simulations performed in the same work indicated that when NPs are pressed into the oil side of the interface, they seem to be strongly overcharged by the counterions.

**Figure 3:**
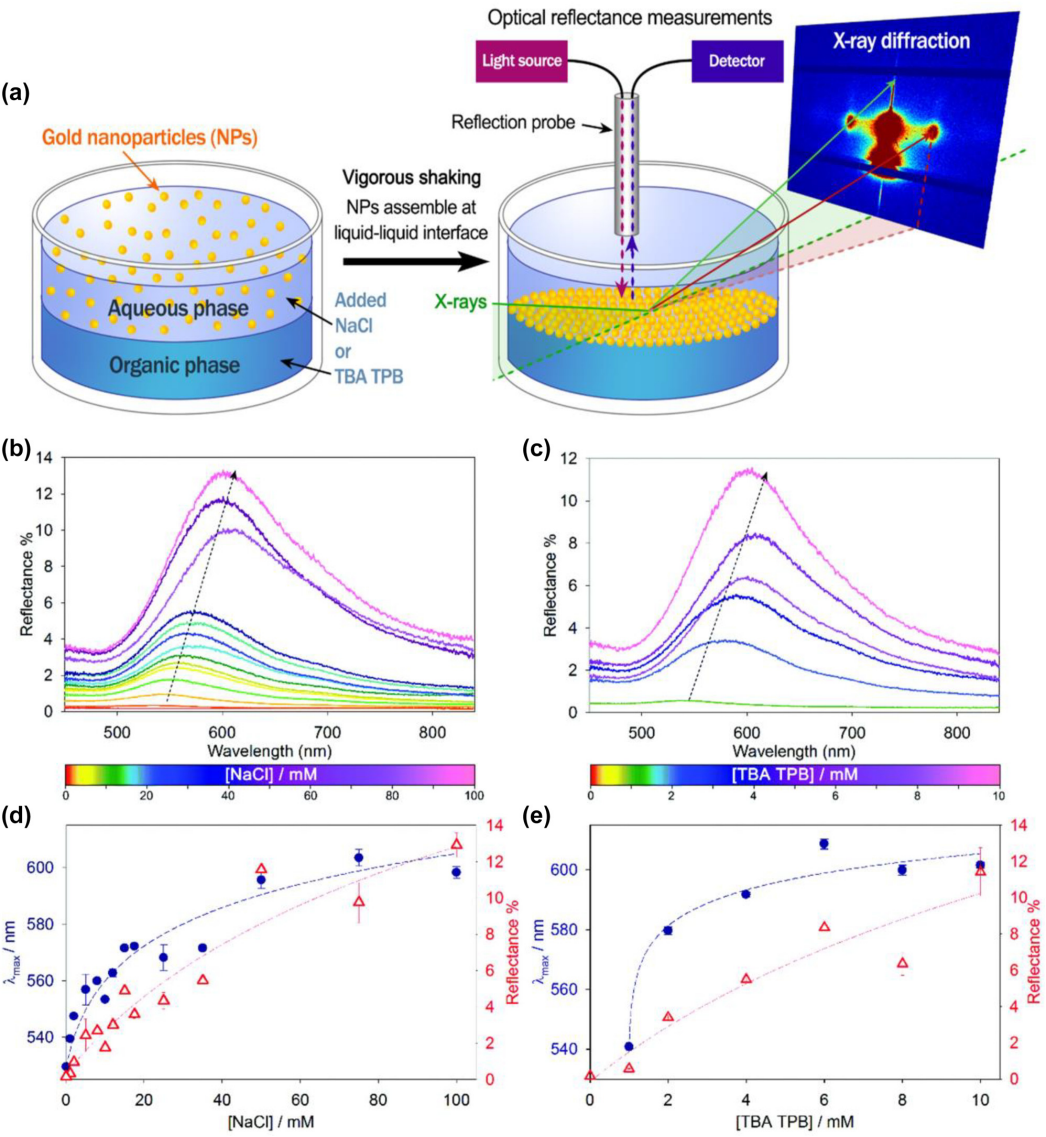
(a) Concept of optical reflectance/X-ray diffraction for detection of an array of nanoparticles, spontaneously adsorbed at an oil/water interface controlled by the concentration of either inorganic electrolyte dissolved in water or of organic electrolyte dissolved in oil. Reflection spectra from the NP arrays at 1.2 Dichloro**e**thane (DCE)/water interface varying with the concentration of NaCl in water (b) or Tetrabutylammonium-Tetraphenylborate in DCE (c). (d) and (e) are the corresponding values of the reflection maxima (red triangles) and wavelengths of the maxima (blue dots). Reproduced from [97] with permission from the Royal Society of Chemistry.

That work inspired the Imperial team to initiate and lead a study, crucial for better understanding the assembly of larger particles that are optically responsive (Figure 3(a)). In that study [97], both the optical reflectance and x-ray diffraction from NP arrays spontaneously adsorbed at an LLI were investigated. The array density was controlled by varying the electrolyte concentration *either in the aqueous* or *in the organic phase.* The experiments were performed at the Dimond Light Source in the UK. Importantly, the X-ray diffraction and light reflection measurements were executed *at the same time in the same cell*. It should be noted that it was not an electrochemical cell, and the light reflection was not large, since without the assistance of an applied voltage highly dense arrays did not form. Furthermore, the AuNPs used were of intermediate size, 12.8 nm also limiting the total reflectance. Nevertheless, the reflection spectra taken at normal incidence of light to the LLI were sufficiently strong and hence detectable. X-ray diffraction confirmed an almost ideal hexagonal structure of the arrays, as well as the expected decrease in the inter-NP distance with the increase of electrolyte concentration. For each electrolyte concentration, and thereby the inter-NP separation in the array determined experimentally, it was possible to calculate virtually without adjustable parameters the whole reflection spectra and compare them with the experimentally measured ones. The results have shown excellent correspondence between theory and experiments, thus proving that the basic ideas underpinning the whole project were correct.

Several factors are important for obtaining the mirror effect, such as the density of NP arrays, and of course, the material and the shape of NPs; both of them determine the spectrum: the material determines the position, height and breadth of the plasmon resonance; the proximity of NPs determine the redshift of reflectance maximum. But one factor that is crucial for the overall intensity of reflection is the size of the NPs. The smaller the NPs, the smaller the reflectivity. Girault’s group [98] has studied systematically the size effect, controlling the population of LLI with NPs of different sizes by direct addition of NPs to the interface (the method, however, did not exclude the formation of multilayers of NPs, especially for larger NPs). It was found, not unexpectedly, that for NPs above 60 nm in diameter, scattering effects start to interfere with the reflection (the latter goes down with a further increase of the size of NPs (see Figure 4 of Ref. [98]). There is, thus, an optimal NP size for an “ideal” mirror, as the wave-length must be much larger than the diameter of NPs, to “see” the “NP array” as a smooth continuum film. At the same time, if the NPs are too small, their “plasmonic mass” – the number of free electrons in them, and correspondingly the mode’s “oscillator strength” – will also be too small, and the presence of such NPs at the interface will be invisible in linear optics. Interestingly, in that study, the NP population at the interface was controlled by measuring the electronic conductivity across the interface (electrons presumably tunnelling along the ligands between the NPs). Therefore, in Ref. [98] they also plotted the reflection signal as a function of the estimated population of NPs.

**Figure 4:**
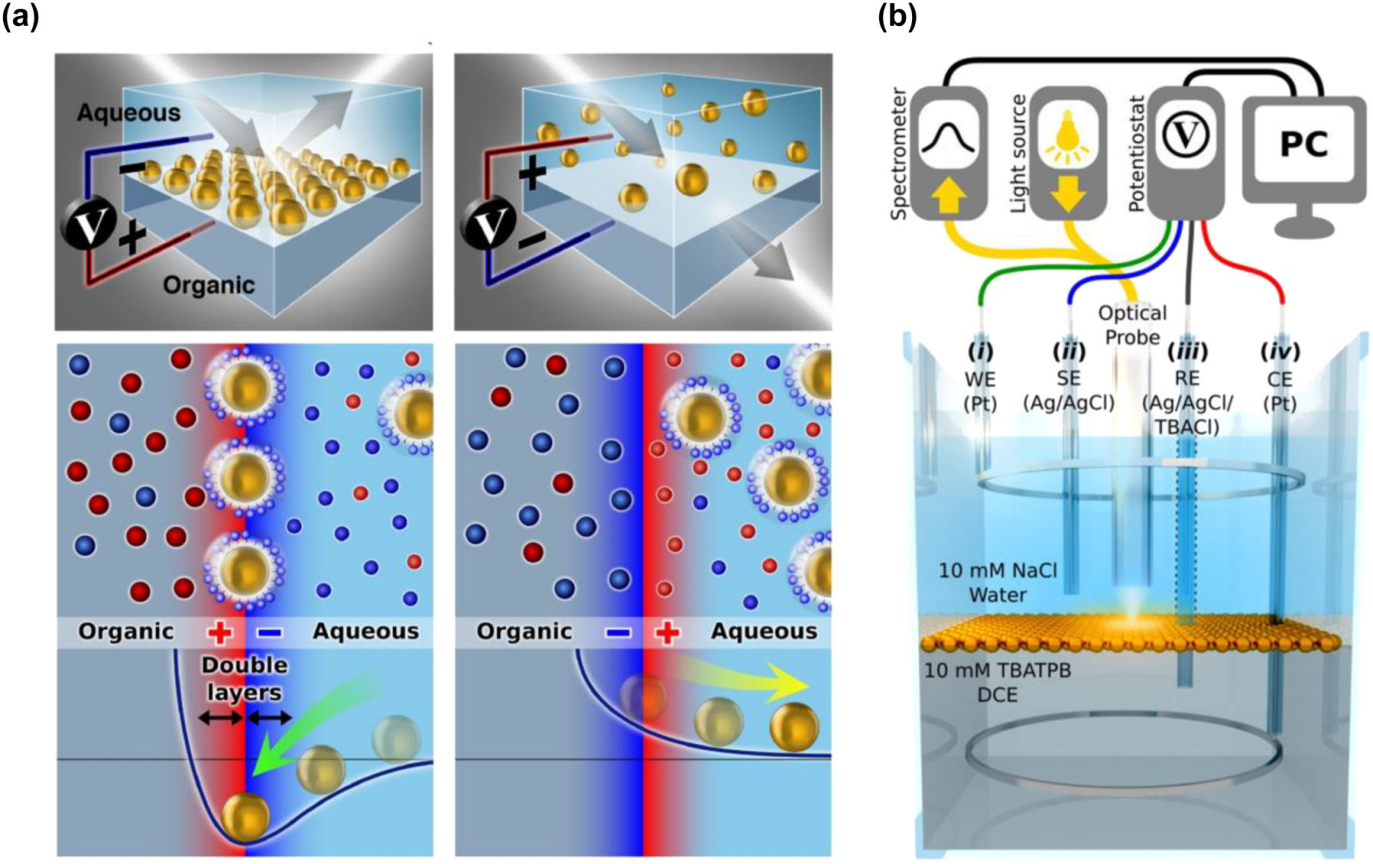
(a) Schematic of electrosorption or electrodesorption of negatively charged AuNPs of arrays assembling or disassembling from the ITIES. The array density is controlled by an applied voltage across the ITIES. (b) Schematic of the ITIES electrochemical cell with an optical probe. The probe transmits light onto the interface and collects reflected light to the spectrometer. The potentiostat is connected to the cell through (i) working (WE) and (ii) sense (SE) electrodes in the aqueous phase, and (iii) reference (RE) and (iv) counter (CE) electrodes in the organic phase. All electrodes are protected with glass capillaries; WE and CE have ring terminals. Reproduced from [99] with permission. Copyright © 2017, Nature Publishing Group.

As mentioned, the experiments at the Diamond Synchrotron Light Source did not and could not yet involve any elements of electrovariability. It thus remained to demonstrate the latter in an ITIES *electrochemical* cell. The corresponding opto-electrochemical setup was later built within the J.B. Edel and A. Kucernak laboratories. The experiments were performed with 16 nm AuNPs using aqueous and DCE solutions along with their respective electrolytes, Na^+^Cl^−^ in the aqueous phase and TBA^+^ TPB^−^ in the organic phase. The concept behind the experiment along with a custom-built electrochemical cell used is shown in Figure 4. The experimentally measured reflection spectra at normal incidence, obtained for each voltage were treated with the theory by fitting first the wavelength of reflection maxima and their height, thereby extracting the information on the average inter-NP separation (as there was no independent X-ray data available for it), and then calculating the whole spectra. It was full success again: the calculated spectra where practically exactly like the measured ones. This was the first realisation and characterization of an *electrovariable* mirror at an electrochemical interface [99].

Edel and Montelongo recorded a video, showing how it operates: https://www.youtube.com/watch?v=68J0yLvrvJE. The video has been viewed on the web more than 24,000 times, and discussed on many websites, see, e.g. https://www.photonics.com/Articles/Tunable_Nanoparticle_Layer_Switches_Between/a62514. It shows how a tiny, just 0.5 V, variation of voltage can change the interface from a “mirror” to “window” mode. It displays either reflection of a coin positioned above the interface onto which the light is shone where AuNPs form a dense array. When the voltage is switched to push NPs away from the interface back into the bulk of the aqueous phase, the interface becomes transparent, and a £10 banknote positioned underneath the interface becomes visible.

**Figure 5:**
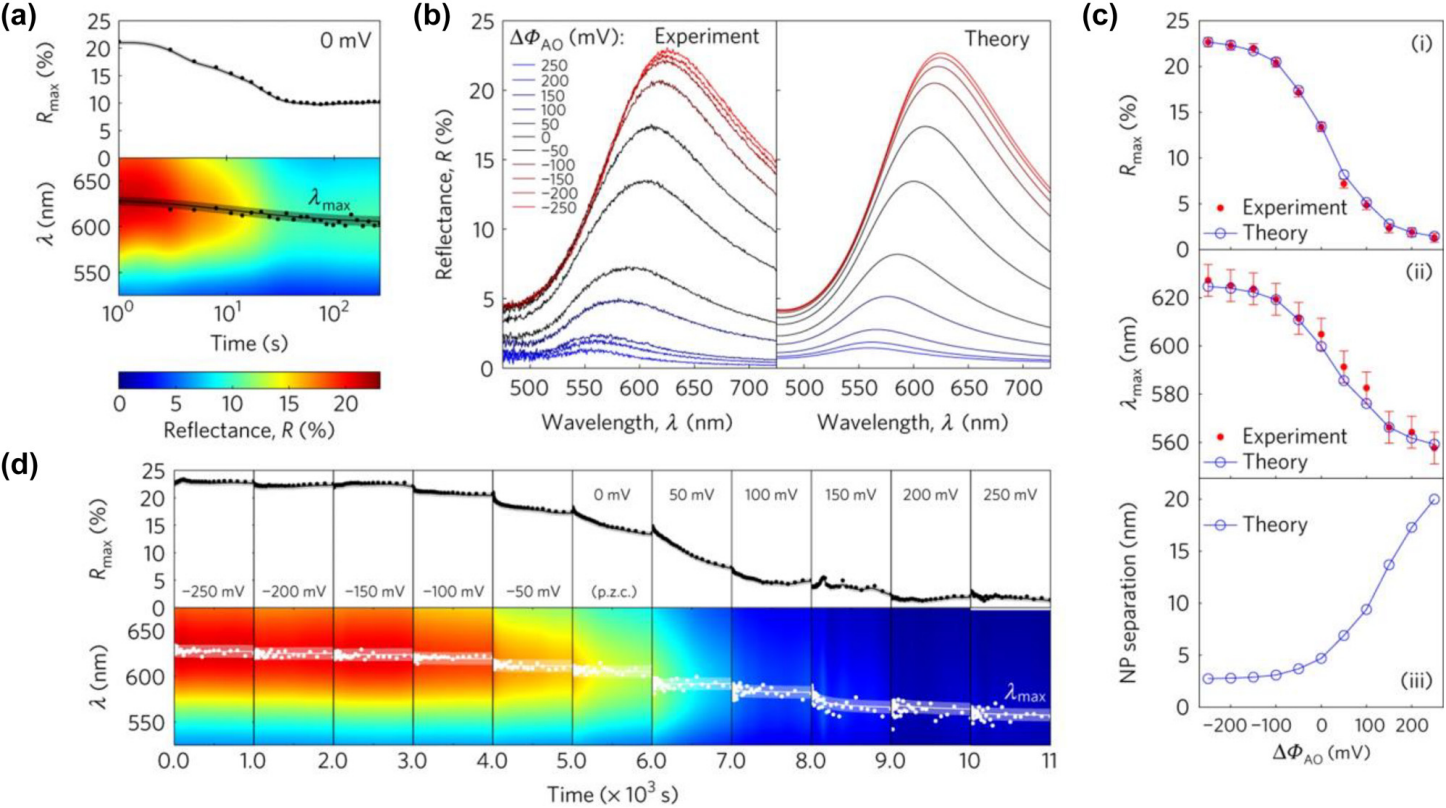
(a) Evolution of peak reflectance *R*_max_ and peak wavelength *λ*_max_ of the NP mirror. The experiment was conducted in a two-step process: first, the NP mirror was assembled at a negative polarization (−250 mV) of the aqueous phase relative to the organic phase. Then the potential was increased to the p.z.c. and kept at that value for 200 s—the spectra were recorded until they stabilized. (b) Optical reflectance spectra were recorded at steady state for different applied electric potentials: experimental (left panel) and theoretical (right panel). (c) Comparison between the experimental data (red filled circles) and theoretical calculations (blue open circles connected by a line) for (i) *R*_max_ and (ii) *λ*_max_ for different applied potentials; (iii) shows variation of the inter-NP separation, fitted for the theory to reproduce the experimental reflectance spectra, with applied voltage. (d) Steady states were observed when the NP mirror was disassembled in a multi-step process, starting from a fully assembled layer to a state when the majority of NPs leave the interface. Each step, corresponding to the indicated voltage, lasted 1000 s, after which the spectra stabilized. ITIES: 10 mM NaCl aqueous/1,2-DCE + 10 mM TBATPB organic phases. Reproduced with permission from Ref. [99]. Copyright © 2017, Nature Publishing Group.

From branding to science: the measured spectra (some of which we show in Figure 5) where excellently reproduced by the theory.

**Figure 6:**
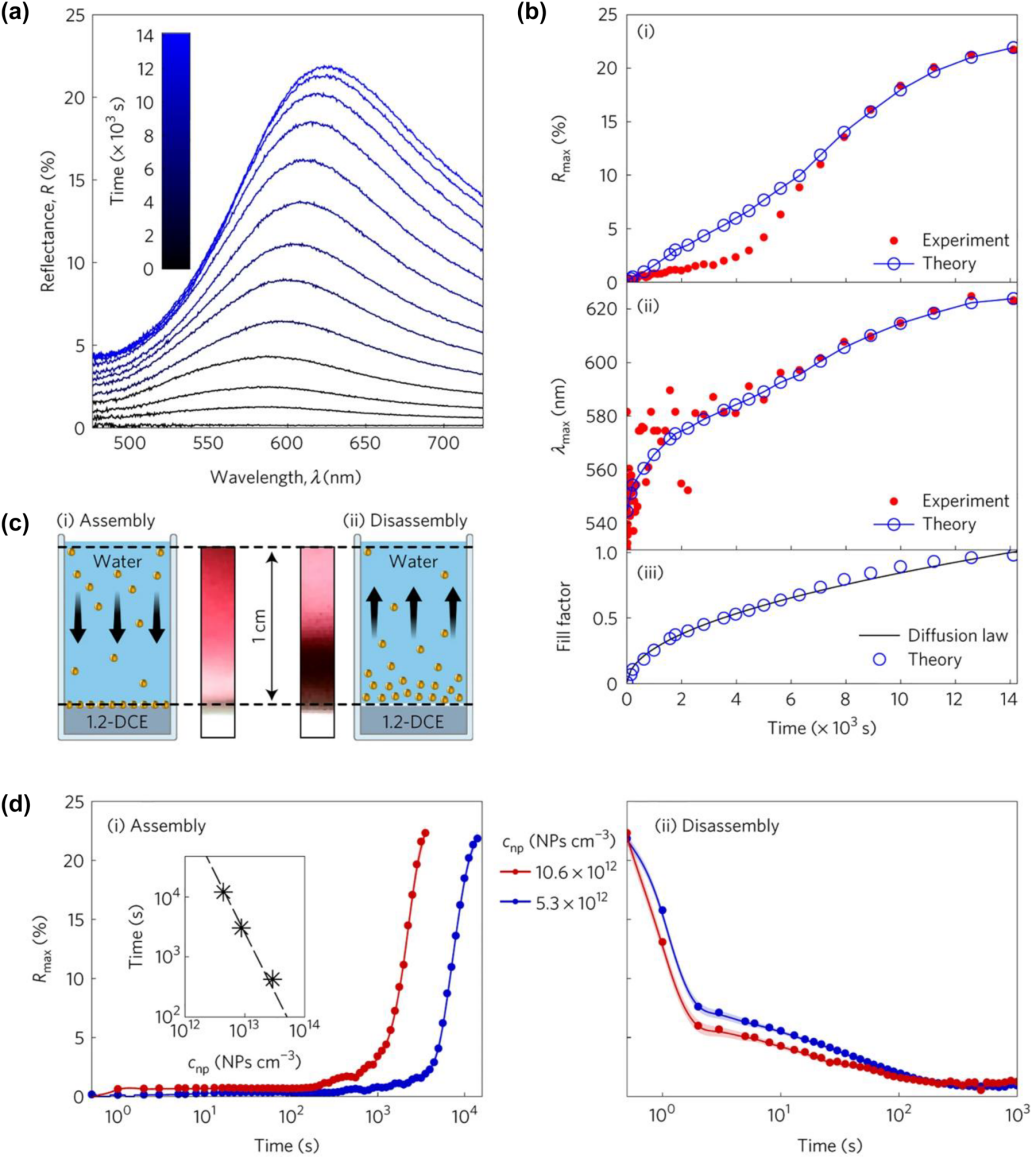
(a) Optical reflectance spectra observed during the assembly process in equally spaced time steps ranging from 1 to 14,116 s at the potential bias −200 mV (water relative to organic). (b) The peak reflectance *R*_max_ (i) and peak wavelength *λ*_max_ (ii) measured during the assembly process (red filled circles) are compared against their theoretical estimates (blue open circles connected by a line). (iii) The time dependence of the fill factors—the coverage of the interface by NPs (blue open circles) obtained from theoretical fitting are then compared against those obtained from the diffusion-limited adsorption (equation (3)) law (solid black curve), with a fitted value of the diffusion coefficient, eventually coinciding with 1.53 × 10^−7^ cm^2^ s^−1^ reported in ref. [102] and the bulk NP concentration *c*_np_ = 5.3 × 10^12^ NP cm^−3^. (c) Cartoons of assembly (i) and disassembly (ii) process “viewed” from the side of the container; the middle colour gradients are real photographs of the same view taken during the experiment, with the ITIES at the bottom of the images. The latter correspond to density distributions of NPs in the aqueous phase during assembly (i) and disassembly (ii) after a period of 4 h. (d) Evolution of reflectance peak *R*_max_ during assembly (i) and disassembly (ii) of NPs for two different concentrations. The potential applied was −200 mV for assembly and 200 mV for disassembly. The scaling of the adsorption time as 1/*c*_np_^2^ is well seen in the inset of (i) plotted for three concentrations of NPs −5.3 × 10^12^, 10.6 × 10^12^ and 35.4 × 10^12^ NPs cm^−3^. ITIES: 10 mM NaCl aqueous/1,2-DCE + 10 mM TBATPB organic phase. Reproduced with permission from Ref. [99]. Copyright © 2017, Nature Publishing Group.

The video, as spectacular as it is, had to be dramatically sped up, because those experiments deliberately avoided dielectrophoresis [100, 101] which could otherwise drive charged NPs to the interface through the aqueous bulk. The static applied voltage cannot do it. Indeed, in the absence of electrical current across the interface, the electric field in the bulk of each phase is screened, and both the aqueous and oil bulks stay electroneutral. The electric field in this system is concentrated only within the electrical double layers on either side of the interface and at the electrodes. Thus, when the aqueous phase was polarized more negatively, increasing thereby the potential wells that trap NPs at the interface, NPs in the bulk “did not know” anything about it. They would learn the difference, only when they get close to the LLI, and they could reach the interface, to get trapped there, only via random diffusion from the bulk, which is a slow process. Compare yourself reaching the point of interest in a historical town with a guide or by random walk… In the initial study of Ref. [71], after the corresponding voltage jump, the formation of the mirror took hours but took place at a rate exactly as the theory predicted (see Figure 6).

Indeed, a simple estimate shows that the diffusion time for NPs to reach the interface to form a monolayer is inversely proportional to the square of the bulk concentration, *c*, of NPs [99]. Increasing their concentration through the increase of the number of NPs would not, however, be a good idea, because the aqueous solution in which such NPs will be dissolved would get heavily coloured – it will look like Bordeaux wine. But one can reduce the thickness of the aqueous phase, maintaining a minimal number of NPs (just enough to cover the monolayer at the interface). This has been tested experimentally, and the 1/*c*_np_^2^ law for the switching time was confirmed [99]. This opens the door to building *electrochemical microcells*, without invoking dielectrophoresis (involvement of the latter is possible, but it would have made practical implementations of such cells more complicated and energy-consuming). Estimates show that properly designed microcells could deliver millisecond switching time between window and mirror modes.

Such optical switches can be used in various applications, but the realization of a dream to use them in the building industry – switching windows to mirrors in skyscrapers, saving energy on the air conditioning when offices are not occupied, as first spelt out in Ref. [7], would require vertical orientation of the interface. From this angle, ITIES do not look… too practical. Electrochemical cells with vertical ITIES have been created from the early days of ITIES in the laboratory (see papers cited here), but no one tried to upscale them.

Another research team in the UK, Dryfe’s group in Manchester, has been intensively studying the optical properties of NP arrays at ITIES, including voltage control over those properties. For instance, they have reported electrotuning SERS signals at the ITIES via a voltage-controlled population of AgNPs at the interface [103]. In this context, we refer the reader to a recent comprehensive review article by Booth and Dryfe [104].

## 5 Controlled self-assembly of nanostructures at solid|liquid interfaces

The first thought of the Imperial team was to create similar optical switches by adsorbing/desorbing NPs from electrolytes onto/from transparent solid electrodes, such as ITO, ZnO, or graphene on a glass substrate. Corresponding calculations have been performed showing very promising results. But this idea has not yet been experimentally realised. In the meantime, a more conventional (for electrochemistry) prototype of such a system was considered, based on a metal/electrolyte interface.

**Figure 7:**
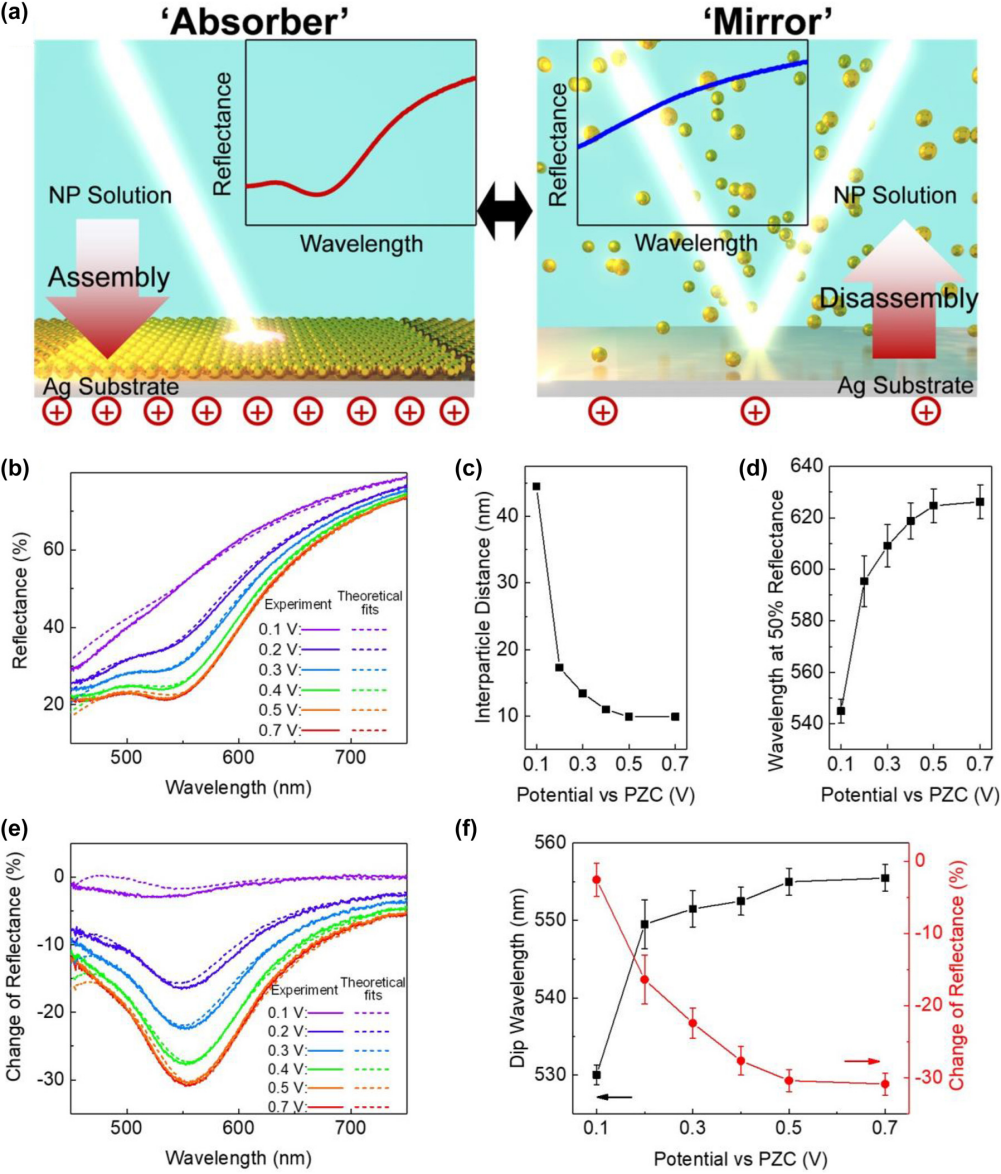
(a) A schematic of the voltage-controlled mirror-absorber switch at the electrode/electrolyte interface. For an applied voltage that cannot keep negatively charged gold NPs at the interface, the particles leave the surface, and the incident light reflects from the silver mirror (right); for more positive voltages (left) NPs adsorb at the interface which results in broadband quenching of reflection. (b)–(f) Optical reflectance from mirror modified by electrosorption of Au NPs, on a silver electrode, controlled by the electric potential. (b) Experimental (solid) and theoretically fitted (dashed) reflectance curves of disassembling NPs on TiN/Ag in 60 mM NaCl under 0.1–0.7 V versus PZC. The experiments start from fully assembled NPs/TiN/Ag at 0.7 V versus PZC, with gradual disassembly by stepwise reducing the potential to 0.1 V versus PZC. The wavelength at 50 % reflectance (c) and the fitted interparticle distances (d) for NPs assembled on TiN/Ag at different potentials (as derived from data in Panel b). (e) Experimental (solid) and theoretically fitted (dashed) curves of the change of reflectance of NP/TiN/Ag with respect to the pristine TiN/Ag substrate under different indicated potentials. (f) The wavelengths (black squares) and the change of reflectance (red dots) at the reflection dip, as derived from the data of Panel e. Reproduced with permission from Ref. [109]. Copyright © 2018 American Chemical Society.

A purely theoretical analysis, confirmed also by full wave simulations, has shown an interesting effect, when NPs get electrosorbed/electrodesorbed on/from a metallic mirror electrode [92] (Figure 7). Without NPs adsorbed on the surface, the latter provides classical wide band reflection spectrum of that surface. But as predicted by the theory, the formation of dense monolayer of adsorbed plasmonic NPs of sufficiently large size leads to a *broad-band quenching* of the reflection. The wavelength of the reflection dip is centred again at the wavelength of the plasmon resonance of individual NPs, moving slightly to the red with the densification of the array.

This effect was not broadly known. All aspects of it were clearly described in Ref. [92], but it was also predicted within an earlier version of the effective medium theory [105] (substantially improved in Ref. [92]), and by Truong and de Dormale [106], who used a different theoretical method. In another configuration (adsorption of AuNP on thin gold film, that allowed studying light reflection from the adsorbed NP-array side as well as shining light onto the other side of the film, thus probing plasmon resonances by evanescent wave excitation) an effect of similar nature was experimentally studied by the group of David Smith [107]. Moreover, in an often overlooked early paper of the Liverpool group, led by David Schiffrin, signatures of a similar effect have been seemingly observed in the electroreflectance signal from the bi-layer of 5 nm AuNPs on a functionalized gold electrode [108].

Within the acquired potential window, the array was never denser than the one corresponding to 5 nm inter-NP surface-to-surface separation. But even then, as was found in a later paper [110], for large NPs (40 nm in diameter), almost 100 % quenching of reflectance was observed at the wavelength of reflection minimum. All the experimentally obtained spectra were fitted to the theory, through the wavelength of the reflection dip and its depth, with one fitting parameter – the average distance between NPs. Thus, the average inter-NP separation was retrieved for each applied electrode potential. Then, again, using the values of this key parameter of the theory, the whole spectra were calculated, reproducing amazingly well the experimental results.

**Figure 8:**
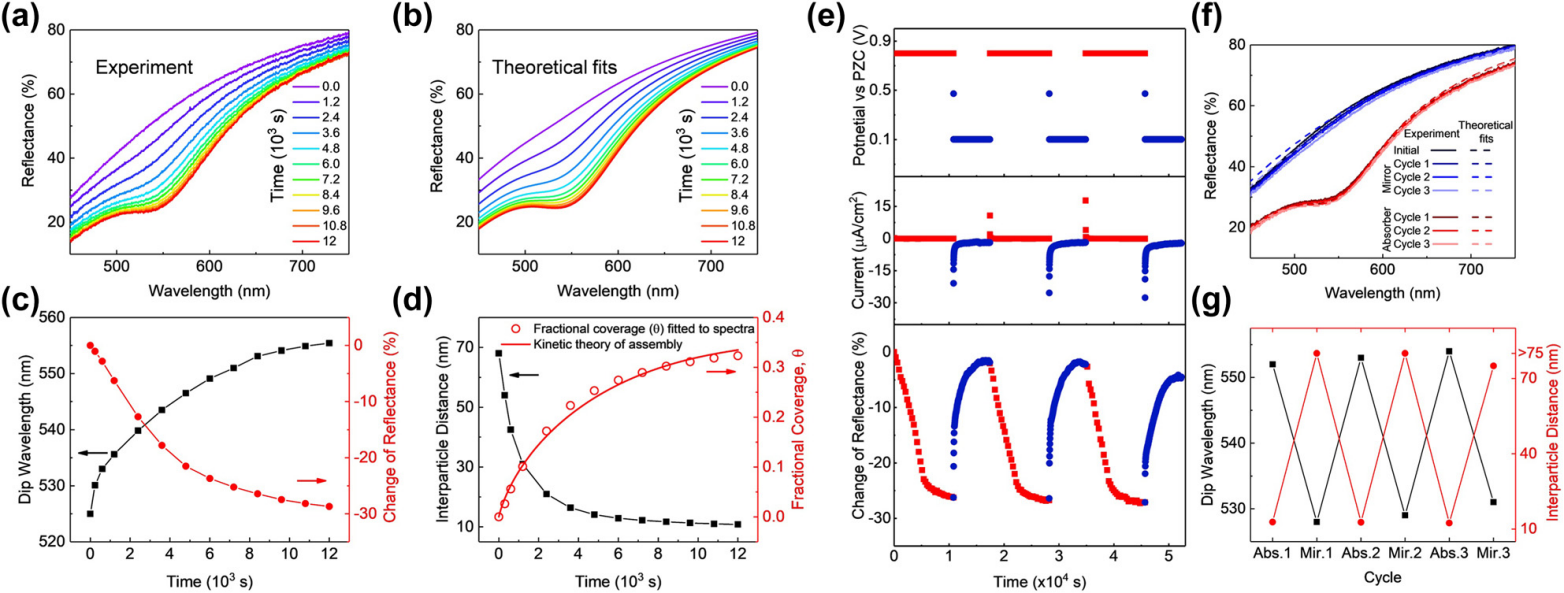
Kinetics of the NP assembly triggered by electrode potential. Experimental (a) and theoretically fitted (b) time-dependent reflectance curves of NPs assembled on TiN/Ag in a solution of 2.2 × 10^12^ cm^−3^ NPs and 60 mM NaCl under 0.7 V versus PZC, starting from pristine TiN/Ag. (c) Corresponding experimental time-dependent wavelengths (black squares) and the change of reflectance (red dots) at the reflection dip (obtained by subtracting the reflectance data for pristine TiN/Ag from Panel a). (d) Time-dependent interparticle distances (black squares); the fractional coverage *θ*(*t*) (red empty circles), as extracted from fitting the theory to reflectance data, and the numerical results from the kinetic adsorption theory (red line), as described in the text. Reversible switching of the system between the “mirror” state (0.1 V vs PZC) and the “absorber” state (0.7 V vs. PZC). (e) Electrode potential over time (top), the corresponding electrochemical transient current (middle), and the change of reflection dip (bottom). (f) The “mirror” and “absorber” spectra at different cycles. (g) The wavelength of the absorption dip and the corresponding interparticle distance (NP surface-to surface separation for the “absorber” and “mirror” states at different cycles. Reproduced with permission from Ref. [109]. Copyright © 2018 American Chemical Society.

As a “side product” of the analysis in Ref. [109], the kinetics of electrosorption/desorption of NPs was studied, Figure 8(a)–(d). As can be seen, electrodesorption, takes much less time [109] (“it takes years to build a skype-scraper, and less than half an hour to destroy it”). Treatment of the data using the theory of kinetic adsorption isotherms [111] shows that the adsorption kinetics is not purely diffusion limited, but there is a barrier for adsorption (and for desorption). Nevertheless, as seen in Figure 8(e)–(g), reproduced from the same Ref. [109], the process of voltage-controlled electro-sorption/-desorption/is reversible.

**Figure 9:**
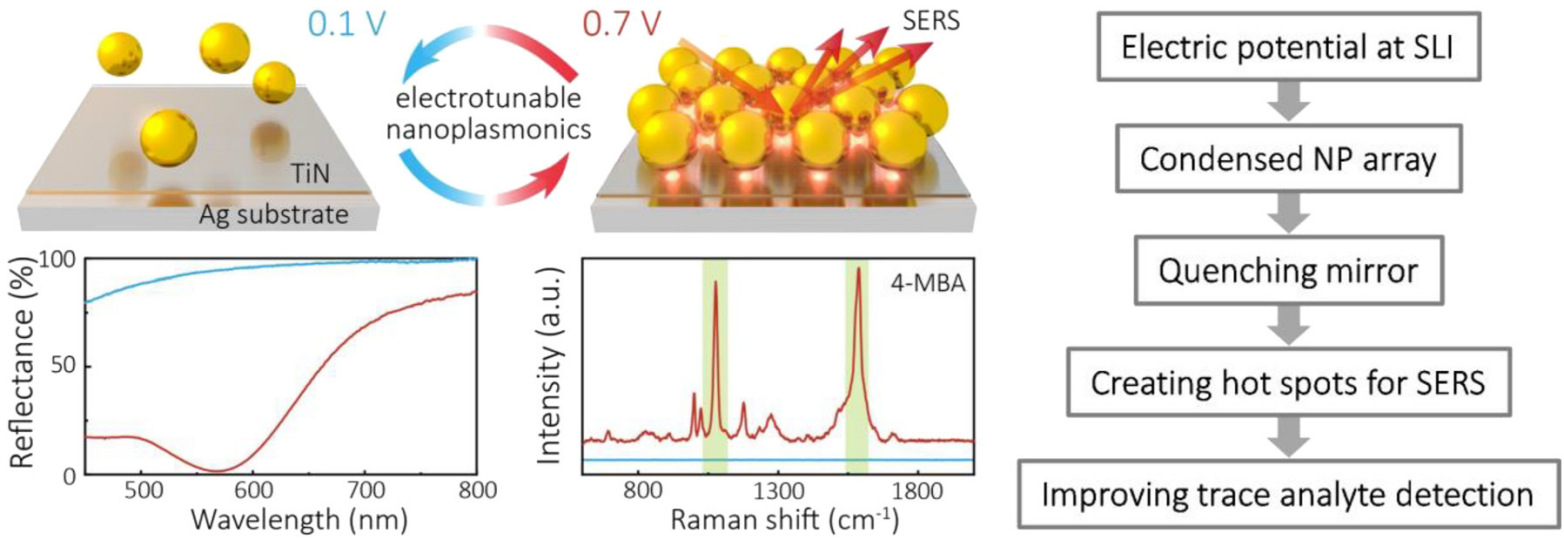
Electrosorption of AuNPs when changing electrode potential by just +0.6 V dramatically changes the light reflectance from that of a pristine TiN-thin-film-covered Ag electrodes (blue) to a broad range quenching of the reflectance signal (pink). Simultaneous measurements of the Raman signal show an equally spectacular change in the Raman signal from mercaptobenzoic acid functionalized NPs: from no signal to a profound Raman fingerprint. Reproduced with permission from Ref. [110]. Copyright © 2019 American Chemical Society.

Encouraged by this success, the Imperial team went back to NP-array-based SERS measurements, not at an LLI, but on a metal substrate. Physically, the space between electrosorbed NPs (still protected against direct contact with the metal substrate by their functionalizing ligands) and the electrode delivers hot spots for electromagnetic radiation, even when the NPs are far apart. When NPs come closer to each other additional hot spots emerge in the space *between* NPs, as well as those *under* the NPs. Thus, the Raman signal from an analyte that can get into the mentioned regions will get enhanced. Those effects have been demonstrated in Ref. [110]. Again, varying electrode potential altered the average distance between the negatively charged NPs; more positive electrode polarisation created a denser array of NPs and amplified the SERS signal. The idea of these experiments and the snapshot of the main result is summarized in Figure 9.

**Figure 10:**
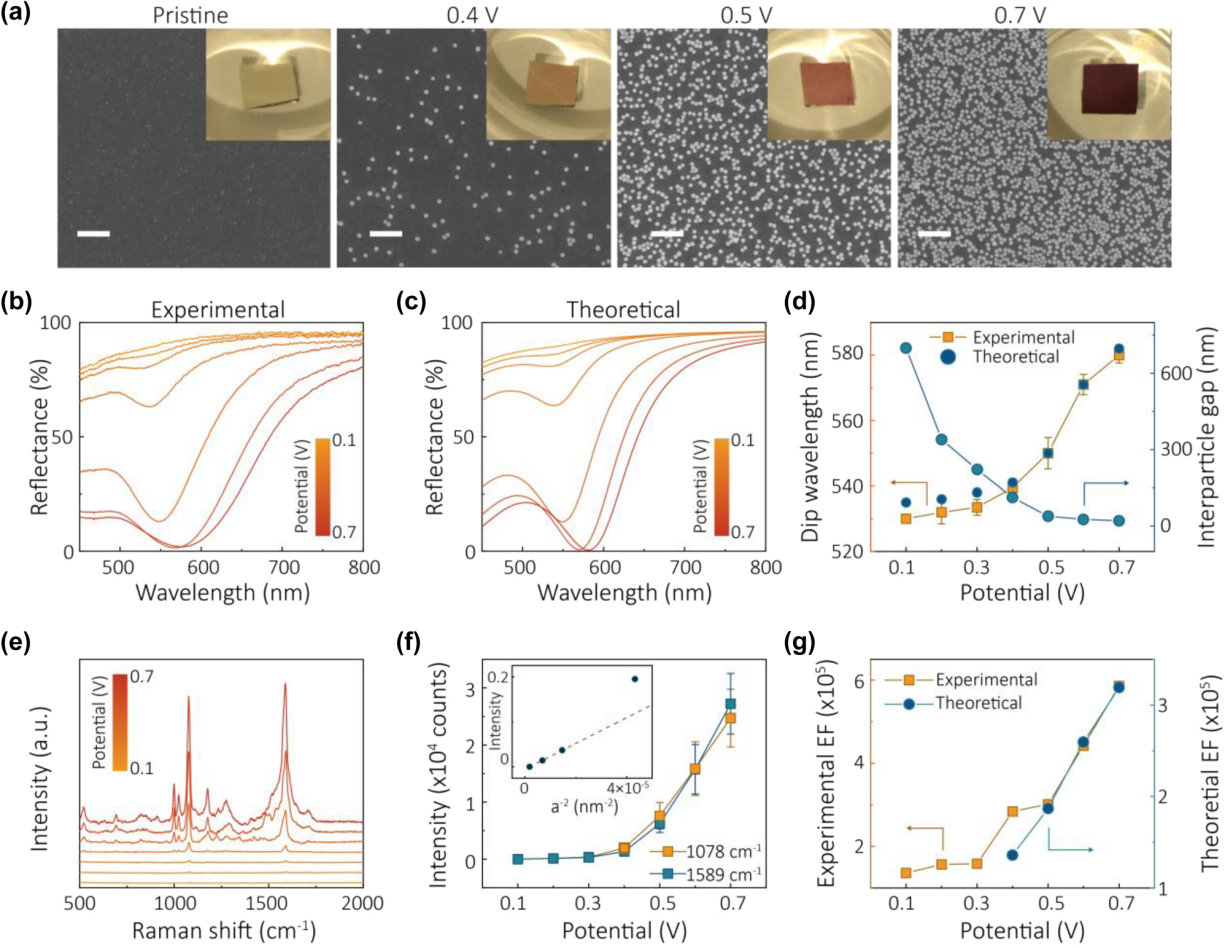
Assembly of nanoparticles by changing the interfacial potentials studied via optical reflection SERS, and scanning electron microscopy (SEM) [110]. (a) SEM of dried samples after NP assembly under indicated potentials; scale bar 200 nm, and digital camera photos (insets) of wet samples after NP assembly on TiN/Ag under indicated potentials that just visualise the dramatic colour change. (b) Experimental reflectance spectra of assembling NPs on TiN/Ag under 0.1–0.7 V versus PZC. (c) Theoretically fitted reflectance spectra of assembling NPs on TiN/Ag under 0.1–0.7 V versus PZC. (d) Experimental and theoretically fitted dip wavelength (orange squares) and the theoretically calculated interparticle gap (blue dots) under 0.1–0.7 V versus PZC. (e) Experimental Raman spectra of MBA from the NP array on TiN/Ag under 0.1–0.7 V versus PZC. (f) The intensity of characteristic Raman peaks of 4-MBA at 1078 cm^−1^ (orange) and 1589 cm^−1^ (blue) under 0.1–0.7 V versus PZC. Inset shows the 1078 cm^−1^ Raman peak intensity (I) versus the inverse of the square of the interparticle distance (a^−2^), extracted from fitting the reflectance data to the theory (cf. panel d), in the interval of 0.1 V between 0.1 and 0.4 V versus PZC. (g) Experimental (orange squares) and theoretically calculated (blue dots) SERS enhancement factor (EF) under 0.1–0.7 V versus PZC. The theoretical EF was not calculated below 0.3 V versus PZC as the gaps are too large to return converging results. Reproduced with permission from Ref. [110]. Copyright © 2019 American Chemical Society.

The signal increased proportionally to the increase in the number of NPs on the surface but only when NPs were on average still far from each other. This must be so, if the hot spots lie between the NPs and the electrode. But further densification of the NP array has shown a stronger increase of the Raman signal from the analytes with the array density. It was thus a clear demonstration of the emergence and crucial role of hot spots not only underneath NPs but also in between NPs. Plasmon-resonance wise, NPs start to “talk to each other”! In this work [110] the Raman measurements were complemented by simple reflectivity measurements, as a result, the latter made it possible to retrieve the average distances between NPs, and thus it was possible to correlate NP spacing to the enhancement of the Raman signal. Agreement between theory and experiments is spectacular. Figure 10 demonstrates this, showing the results of systematic studies along these lines.

Remarkably, close to the frequency of plasmon resonance for 40 nm AuNPs at approximately 30 nm surface-to-surface separation one reaches full quenching of reflectance (see Figure 10(b)), which is validated by the theory (Figure 10(c)). Panels (d) and (g) give evidence that, as we have just mentioned, the intensity of the Raman signal does not grow proportionally to the density of NP array, but much faster, which indicates the important role of the hot spots in the interparticle spacing, and not only those between NPs and the electrode. The noted “conversation” between NPs starts when the NP surface-to-surface spacing is already 100 nm (2.5 times their diameter!) and it of course becomes more pronounced when they come every little bit closer.

A spectacular feature of Figure 10(b) and (c), is that at the achieved closest 20 nm gaps between NPs one observes almost *complete* quenching of reflectivity in the domain of wavelengths between 550 and 600 nm. At the same inter-NP distances, as expected and observed, one gets the highest SERS signals from NP-functionalizing ligands.

**Figure 11:**
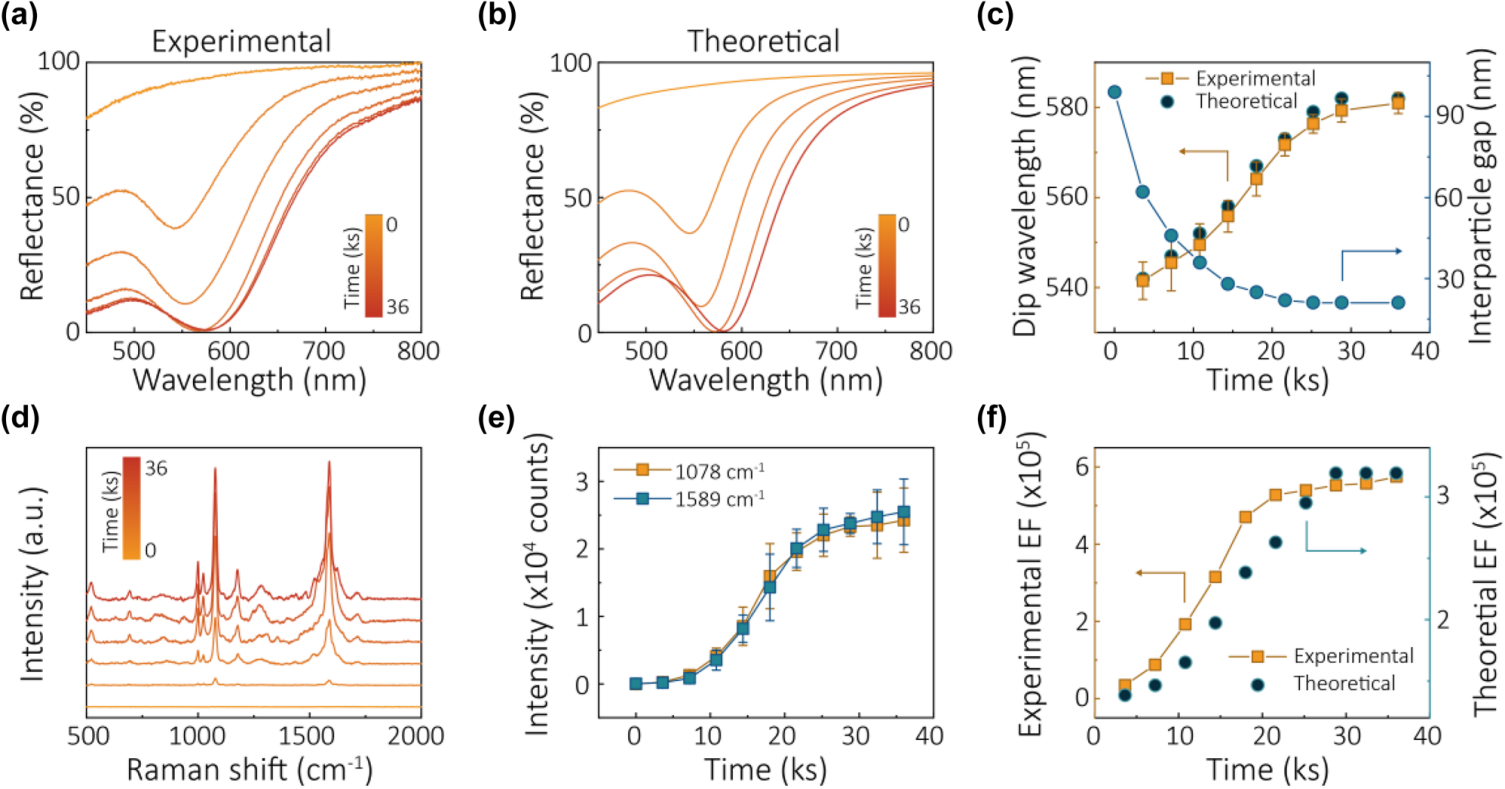
Kinetics of voltage induced self-assembly of NP arrays [110]. (a) The time-dependent reflectance spectra of AuNPs electrosorbed on TiN/Ag in 20 mM PB, 20 mM LiCl NP solution, under 0.7 V versus PZC, starting from pristine TiN/Ag. (b) The theoretically fitted time-dependent reflectance spectra of NPs assembling on TiN/Ag. (c) Time-dependent experimental, theoretically fitted dip wavelength (orange squares), and the theoretically calculated interparticle gap (blue dots). (d) Time-dependent SERS spectra of 4-MBA attached to NPs assembling on TiN/Ag in 20 mM PB, 20 mM LiCl at 0.7 V versus PZC, starting from pristine TiN/Ag. (e) Corresponding experimental time-dependent intensity of characteristic SERS peaks of 4-MBA at 1078 cm^−1^ (orange) and 1589 cm^−1^ (blue). (f) Time-dependent experimental (orange squares) and theoretically calculated (blue dots) SERS enhancement factors (EF) for 1078 cm^−1^ under 0.7 V versus PZC, starting from pristine TiN/Ag. Reproduced with permission from Ref. [110]. Copyright © 2019 American Chemical Society.

The combination of reflectivity and SERS allows one to unravel the kinetics of electrosorption of NPs, useful for electrochemical photonics, but also, no less important for colloid science. For instance, one of the curves in Figure 11(c) shows the values of average surface-to-surface separations of NPs in the assembled arrays as a function of time, obtained by fitting the, practically, only free parameter of the theory to the experimental reflectivity data [110]. That curve is the first optically revealed NP’s kinetic electrosorption isotherm.

Recently the optical response of the system composed of an array of plasmonic NPs adsorbed on the surface of stacked nanosheet “hyperbolic metamaterial” have been studied theoretically in Ref. [112]. The response varies between quenched and enhanced reflectivity, depending on the volume fraction of the metallic and dielectric components in the hyperbolic metamaterial. The results of the above-described works [109, 110] are reproduced in the two opposite limiting cases – of a pure metal and of a pure dielectric substrate while predicting novel resonances for intermediate compositions of the substrate. Whereas the metal/dielectric ratio in the hyperbolic substrate cannot be changed in time – for each experiment a new substrate should be fabricated – the density of the adsorbed nanoparticle arrays can surely be controlled in real time in an electrochemical photonic cell, as it was done in Refs. [110]. Indeed, the hyperbolic substrate can also serve as an electrode, with a top nanosheet taken to be metallic. With that option in mind, the effect of the array density on the optical response of such systems was systematically studied, which could be later verified experimentally. The manifestation of these findings in a hyperbolic-Fabry–Perot cell are also explored in that paper.

**Figure 12:**
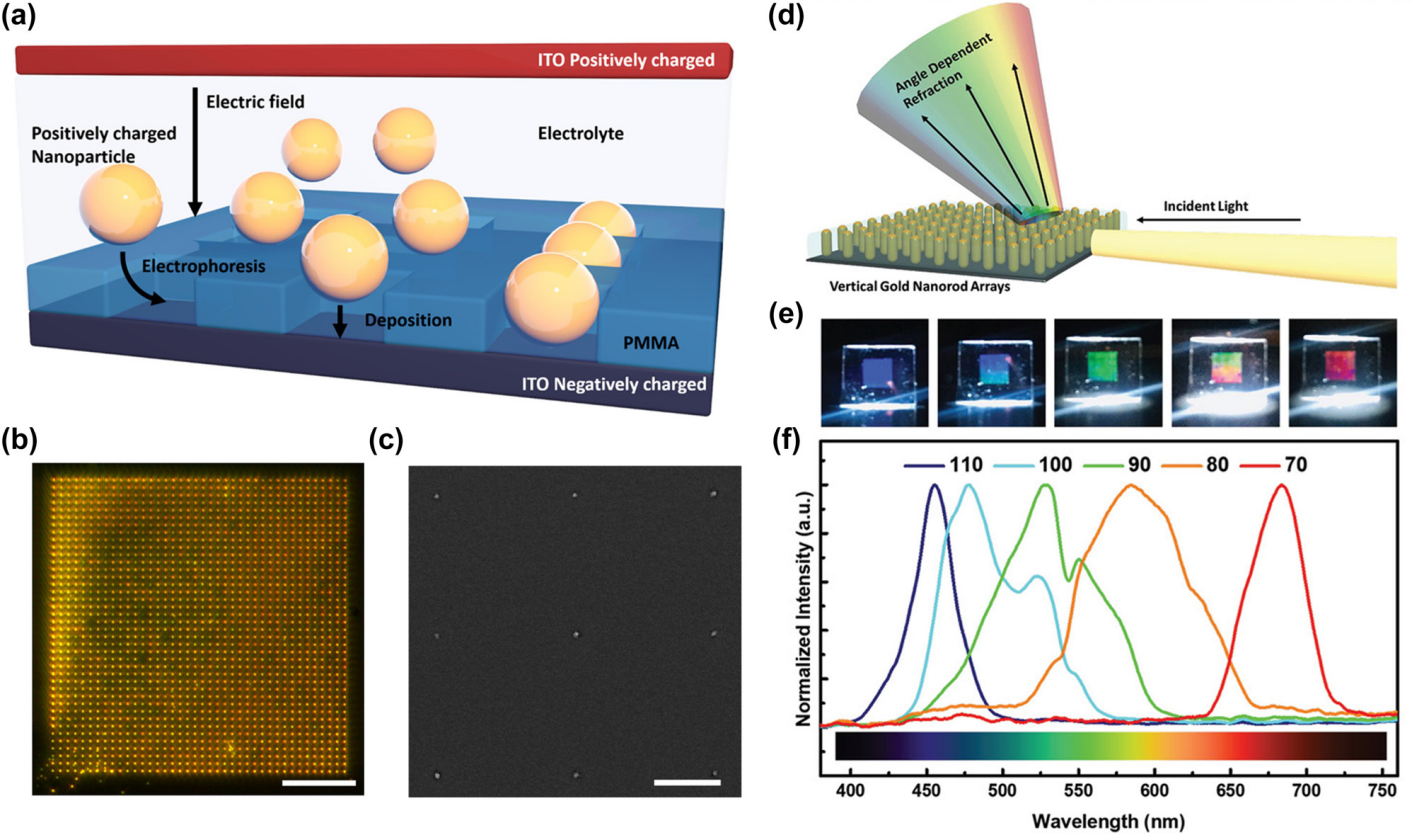
(a) Schematic of the electrophoretic deposition process showing nanospheres deposited onto electron beam lithography (EBL) fabricated ITO-PMMA template (dimensions not to scale). A controlled volume of nanoparticle colloidal solution mixed with a controlled concentration of NaCl electrolyte is confined between an EBL patterned ITO-PMMA template and an ITO counter electrode on the top. Nanoparticles are positively charged by coating poly dimethyl-diallyl ammonium chloride (poly-DADMAC). An electric field is generated by applying a potential between the ITO-PMMA template and the ITO counter electrode. (b) Dark-field image of a 40 × 40 box array filled with 110 nm gold NPs. (c) Scanning electron microscope image of several gold NPs (scale bar: dark-field images, 40 μm; scanning electron microscope images, 2 μm). (d) A schematic of the measurement setup used to collect the scattered light spectrum from the vertical gold nanorod array. (e) Digital pictures of the 4 mm × 4 mm vertical gold nanorod array at different viewing angles from 110° (left) to 70° (right). (f) Normalized refracted light spectra of a vertical gold nanorod array for different collection angles. (a)–(c) Reproduced with permission from Ref. [115]. Copyright 2018 American Chemical Society. (d)–(f) Reproduced with permission from Ref. [118]. Copyright 2020 Wiley‐VCH GmbH.

As we have already mentioned, another effective approach in controlling and warranting the faster assembly of NPs at soilid|liquid interfaces is electrophoresis [113]. Initially, the electrophoretic deposition was largely focused on the assembly of micron-scale particles such as SiO_2_ and polymer beads because, for smaller NPs, Brownian motion can severely hamper particle deposition while double layer effects dominate the NP−interface interactions [114–116]. Recently, the Mulvaney group from the University of Melbourne [115, 117] realized the electrophoretic deposition of gold NPs on solid|liquid interfaces by finely tuning the electric field strength and the electrolyte concentration. An indium tin oxide (ITO) electrode and a second pre-patterned poly(methyl methacrylate) (PMMA)−ITO electrode comprise the electrochemical cell, with positively charged gold NPs dispersed in an aqueous electrolyte between these two electrodes (Figure 12(a)). A DC potential was applied between the electrodes to generate an electric field. The nanocavities in the ITO electrode can be filled by the spherical NPs in 0.2 mM NaCl and 1.5 V/mm field strength. Empowered by electron beam lithography and dark-field scattering spectroscopy, the optical properties of various patterns and interparticle separation at micrometre and even millimetre scale substrates were studied (Figure 12(b) and (c)).

In their later work [118], spherical NPs were replaced by nanorods which demonstrate anisotropic optical properties. Cuboid rather than cubic PMMA nanocavities were printed on ITO electrodes to control the horizontal and vertical orientation of the assembled nanorods array. They found that apart from the strength of the electric field, electrolyte concentration also plays a crucial part in the assembly process. Namely, a low electrolyte concentration is beneficial for fast assembly but also results in more non-specific adsorption on PMMA. Higher concentrations induce strong screening of both the template and particle surface charges, leading to lower particle filling efficiencies and aggregation during assembly. A deuterium-halogen light source was used as a white incident light to illuminate the nanorod array in inspecting the optical properties (Figure 12(d)). The enhancement of different scattering wavelengths can be achieved by modulating the polarization and the incident angle of excitation (Figure 12(e) and (f)) [115, 118]. Not limited to tuneable optical devices, the electrophoretic deposition of NPs shows versatile applications for building SERS sensors [113] and hydrogen evolution [116].

## 6 Further relevant systems using electrochemistry in photonics

First, a few other configurations can be mentioned, based on similar principles. A theory of functioning of Fabry–Perot cell (cavity) that would “trap” light between two semi-transparent electrode plates, and filter the transmission of light through the cell, by controlling the density of NP arrays on the plates through NP electrosorption/desorption from/to electrolyte solution that fills the cavity [119, 120].

Another example involves electro-feeding of the void-space between transparent ZnO electronically conducting nano-columns with NPs or vacating it, subject to the applied voltage [121]. The electrodeposition of metals into well-defined porous structures and dimensions is well known, extending as far as growing ultrathin nanowires in metal-organic frameworks (MOFs) that can function as versatile templates for the growth of metallic nanostructures with precisely controlled shapes and sizes (see e.g. ref [122, 123]). We know how to electrosorb large ionic liquid ions into MOF pores [124]. But, to our knowledge, building columns of individual NPs by physical electrodeposition in pores of large size has not yet been tried.

An interesting opportunity could be offered by anchored systems: changing orientation with respect to the electrode of the metallic (plasmonic) “flat” nano-cuboids linked on one narrow edge to a transparent electrode, with variation of electrode polarization. The calculation shows a remarkable effect of the reorientation of cuboids with respect to the electrode on the overall optical response of the interface [125], with fast dynamics, as not depending on translational diffusion but only reorientation of cuboids. Interesting effects have been theoretically unravelled for controlling the arrays of plasmonic NPs adsorbed on *hyperbolic* metal-dielectric layered composite electrodes. Such systems were theoretically described in Ref. [112]. None of these hypothetical systems has been built and tested. But all the previous experience with their sister systems experimentally studied as described above suggests that they would work as predicted by the theory. Still, in the absence of experimental verification, we are not showing any graphs of the predicted effects, referring interested readers to the cited original papers.

Instead of tuning the NP assembly, electrically changing the surrounding media or even the plasmonic material itself are alternative ways of realizing electrovariable optical properties [126–128]. When an electric potential is applied, large variations in both imaginary and real parts of the complex refractive index can be witnessed in conductive polymers such as polymer poly(3,4-ethylenedioxythiophene): polystyrene sulfonate (PEDOT: PSS), PEDOT: sulfate and polyaniline (PANI) [128–130]. This is because when a positive potential is applied, the polymer is electrochemically doped and oxidized, which results in high carrier density and metallic optical properties. Consequently, the polymer exhibits a strong plasmonic resonance. In contrast, at a negative potential, the carrier density is substantially reduced and the polymer becomes insulating [131, 132].

**Figure 13:**
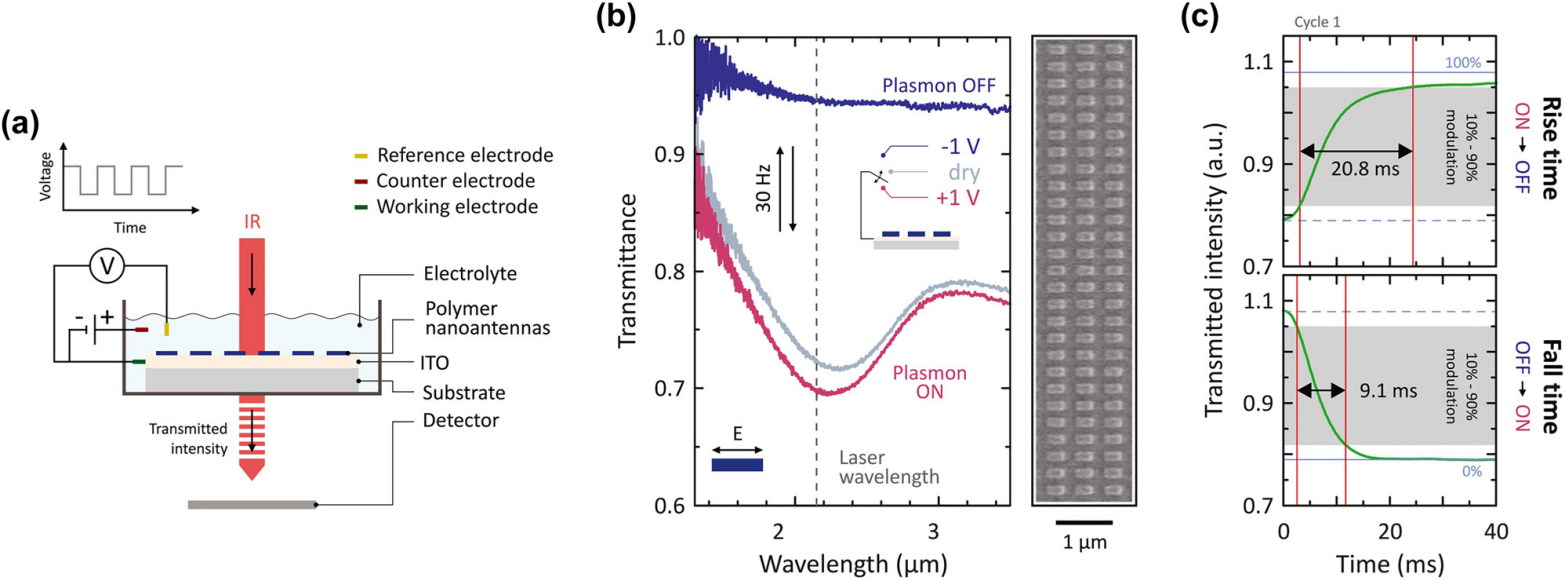
(a) Schematic of the electrochemical cell (three-electrode setup) and optical measurement setup. (b) SEM image and spectral response of metallic polymer nanoantennas (length *L* = 300 nm, width *W* = 160 nm, height *H* = 90 nm, periodicity in *x P*_*x*_ = 500 nm, periodicity in *y P*_*y*_ = 300 nm) for different states. Dry state, pristine (gray): Plasmonic resonance almost completely turned ON. +1 V (red): Plasmonic resonance completely turned ON with highest modulation (polymer fully metallic). −1 V (blue): Plasmonic resonance turned OFF (polymer insulating). Electric field E polarized parallel to the nanoantenna long axis. The dashed line indicates the laser wavelength *λ* = 2.15 μm for (c). (c) Detailed analysis of rise time *τ*_rise_ = 20.8 ms (ON to OFF) and fall time *τ*_fall_ = 9.1 ms (OFF to ON) for the first switching cycle. (a–c) Reproduced with permission from Ref. [131]. Copyright © 2021. The American Association for the Advancement of Science.

**Figure 14:**
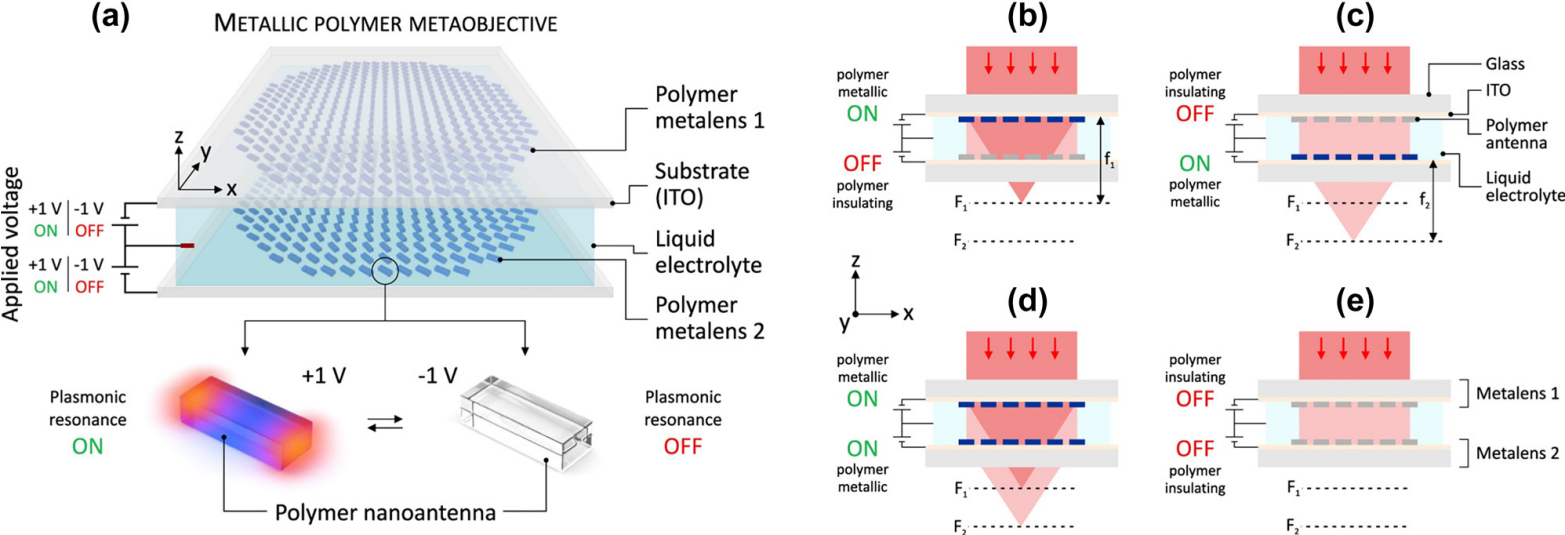
(a) Top: Schematic illustration of a metaobjective comprising two electrically switchable metalenses on ITO (indium-tin-oxide) covered substrates. The metalenses consist of electrically switchable metallic polymer nanoantennas. An electrolyte is used as separation and to allow electrochemical switching. The refractive power of the polymer metalenses is switched ON or OFF on demand via an applied voltage of only +1 V or −1 V, respectively. Bottom: The electrical switching is based on a reversible metal-to-insulator transition of the metallic polymer. A voltage of +1 V turns polymer nanoantennas metallic and their plasmonic resonance ON. A voltage of −1 V switches the polymer nanoantennas into an insulating state and their plasmonic resonance OFF. (b)–(e) Depending on the individual voltage applied to the polymer metalenses, four different states become possible. (b) (metalens 1: ON, metalens 2: OFF): focus at F_1_. (c) (OFF, ON): focus at F_2_. (d) (ON, ON): focus at F_1_ and F_2_. (e) (OFF, OFF): no focus. Reproduced with permission from Ref. [133]. Copyright © 2022, The Author(s).

Using PEDOT: PSS, the Giessen group at the University of Stuttgart demonstrated a fast-switching nanoantenna in the NIR range [131]. The polymer rods, ranging from 90 × 180 × 180 nm to 90 × 180 × 380 nm, were pre-patterned on an ITO substrate through lithography. The electrical switching is performed in an electrochemical cell accommodating a three-electrode setup (Figure 13(a)). When the positive potential was applied, the plasmon resonance was turned on and a dip in transmission appeared at ∼2.2 µm (Figure 13(b)). Such dip disappeared when a negative potential was applied, indicating the turning-off of the plasmon resonance. The switching interval of a full cycle can be as short as ∼30 ms (Figure 13(c)). Based on the metamaterials, the group later proposed an electro-active meta-lens by combining two pieces of the above-mentioned PEDOT: PSS nanoantenna on ITO (Figure 14(a)) [133]. Its static focal length is determined by the pre patterned curvature of the 2D quadratic phase profile of PEDOT: PSS nanorods, while the dynamic focal length is tuned by electric potentials (Figure 14(b)–(e)). These tunable metasurfaces open up the possibility for a new level of electro-optical elements [132].

**Figure 15:**
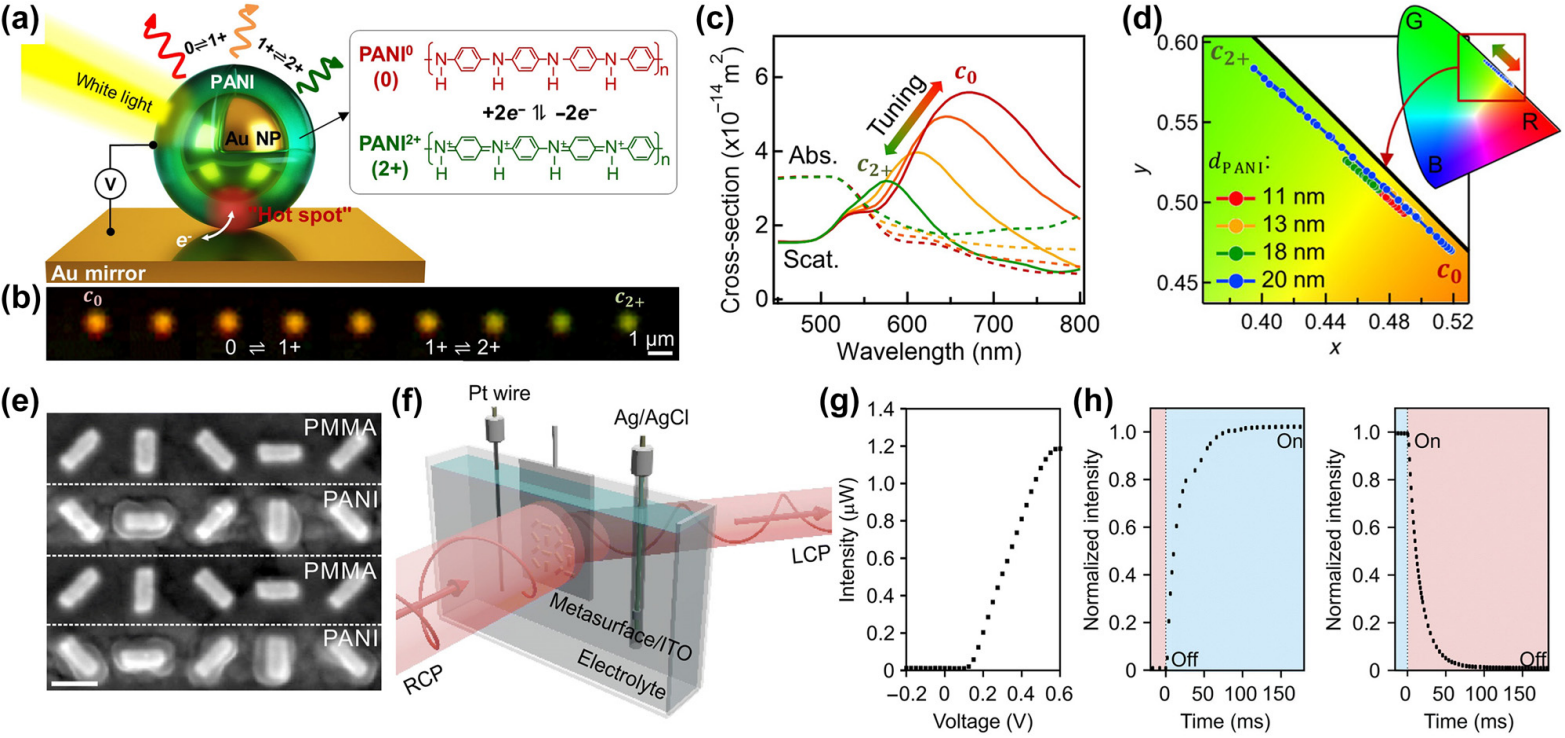
(a) Schematic of an eNPoM, which changes colour as a function of redox state of the thin (0 to 20 nm) PANI shell surrounding each Au NP on Au mirror substrate. Right: Redox reaction of PANI in the gap (PANI^0^, fully reduced; PANI^1+^, half oxidized; PANI^2+^, fully oxidized). (b) Experimental dark-field (DF) scattering images of a single eNPoM nanopixel for different redox states of the PANI shell (left to right: PANI^0^ to PANI^2+^), (c) its corresponding optical scattering (solid lines) and absorption spectra (dashed lines) for different redox states of the PANI shell (red to green: PANI^0^ to PANI^2+^), from numerical simulations. (d) Colour gamut plots for Au NPs coated with different thicknesses of surrounding PANI shell layer (11, 13, 18, and 20 nm). (e) SEM image of the metasurface with the optimized PANI thickness. Scale bar, 200 nm. (f) Schematic of the experimental setup. The metasurface on ITO (working electrode) is immersed into an electrolyte in a glass cell along with a Pt wire (counter electrode) and an Ag/AgCl reference electrode. Right-handed circularly polarized (RCP) light impinges on the sample at normal incidence, and the anomalous transmission intensity is recorded. (g) The intensity of the anomalous transmission as a function of applied voltage. (h) Switching times for the off→on and on→off processes. The rise time is approximately 48 ms (left) and the fall time is approximately 35 ms (right). (a)–(d) From Ref. [134] © The Authors, some rights reserved; exclusive licensee AAAS. Distributed under a CC BY-NC 4.0 license http://creativecommons.org/licenses/by-nc/4.0/”. Reprinted with permission from AAAS. (e-h) From Ref. [135]. © The Authors, some rights reserved; exclusive licensee AAAS. Distributed under a CC BY-NC 4.0 license http://creativecommons.org/licenses/by-nc/4.0/”. Reprinted with permission from AAAS.

Limited by the charge carrier density and mobility of these conductive polymers, the operational wavelengths of these devices locate mainly within NIR range. To make them applicable in the visible range, the combination with noble metal NPs was proposed. The Baumberg group at the University of Cambridge fabricated an Au NP@PANI core–shell structure on a metallic mirror that is electrically tunable within visible ranges (Figure 15(a)) [134]. In response to the increased electrochemical potential from −0.2 to 0.6 V versus Ag/AgCl, PANI experiences a transition from the fully reduced state PANI^0^ to the half oxidized state PANI^1+^ and finally to the fully oxidized state PANI^2+^. The corresponding wavelengths of scattering peaks of such a plasmonic system shift from 642 nm to 578 nm (Figure 15(b)–(c)). The NP to mirror gap was also systematically examined, indicating that a thicker PANI shell results in wider tunable spectral ranges (Figure 15(d)). Such electrically tunable properties highlight its potential applications in colour displays.

Not limited to single NP effects, Kaissner et al. coated PANI onto the lithography patterned nanorods on ITO, creating an electro-tunable metasurface for circularly polarized light (Figure 15(e)) [135]. By shifting the potential from −0.2 to 0.6 V, the transmission intensity can be boosted 860 times, while the switching interval of a full cycle can be as short as ∼83 ms (Figure 15(f)–(h)). To demonstrate the application of the metasurface, two independent pieces were fabricated in parallel, comprising an electrochemical addressable holography. Such a metasurface, as the authors suggested, is applicable in both transmissive and reflective configurations.

Generally, there is a wide class of systems related to electrochemical control of electrochromism. This in itself is an old field, which goes back to the nineteenth century (for review see [136]), based on the idea of modulating light transmission through redox cycling of an electrochromic material. Conventional electrochromic devices show about 80 % transmission modulation with switching speeds of several seconds and colours limited to the naturally arising coloured and bleached states. However, since the beginning of the 21st century, systematic attempts have been made to unlock the full potential of such systems through the exploitation of nanophotonic and nanoplasmonic device architectures, giving the electrochromic effect new facets. A recent article in this journal [137] provided a comprehensive review of all new directions in this area.

## 7 Diversion: electrotunability beyond electrochemistry

The literature here is vast. We will draw for comparison just a few examples.

One class of systems is based on electroactuation, promising for the creation of reconfigurable photonic circuits. For instance, the Australian team of Queensland University has reported a creation of an on-chip high quality microcavity with resonances that can be electrically tuned by voltage variation within 15 V, sub-nanowatt power consumption, and within 0.1 ms response time [33]. This was achieved by integrating nanoelectronic actuation with strong optomechanical interactions that create a highly geometry-dependent effective refractive index across a full free spectral range.

Another interesting option is the use of electroactive two-dimensional plasmonic crystals engineered from GaAs/AlGaAs [138]. It utilizes the complex interplay between surface (Tamm) states, plasmonic defect modes, and the plasmonic crystal band structure in terahertz bandgap devices tunable by gate voltage. Such systems operate however at T < 77 K, but using other materials (e.g. graphene) promises to extend operation to room temperature.

One more direction of research is based on the electrooptical properties of phase change materials [139]. The latter has been studied due to their reversible phase transition, high endurance, switching speed, and data retention. One such material is Germaniumantimony-tellurium, which can be in amorphous or crystalline phases, with distinct optical properties between the two states. It is bistable and nonvolatile and undergoes reproducible phase transition in response to an electrical stimulus, which can be used in tunable photonic devices. Electrotuneable metasurfaces can be built by squeezing a thin film of such material between two film electrodes.

There are many more bright ideas on and realisations of electroactive photonic systems, but reviewing them would lead us too far from the main subject of this article. Many of those systems have their own advantages for specific applications, and their spectral range, but they still have two, not unrelated common features: (i) high speed of response as compared to the conventional electrochemically controlled macrocell systems; (ii) solid state structures avoiding any wet components. In the sections below we will discuss the avenues for electrochemical photonics improvement on those two fronts.

Interfacial optical properties of NP arrays can also be controlled not through applied electrode potentials, but temperature variation in a system that keeps the array together by thermo-auxetic ligands. Such thermally controlled optical switch was based on the self-assembly of poly(N-isopropylacrylamide)-functionalized gold nanoparticles on a planar macroscale gold substrate, which has been built and explored by in Ref. [140]. Shrinking of these ligands with the temperature increase and expansion with temperature decrease affects inter-NPs separation; this effect has been calibrated theoretically and experimentally, in a similar way as in electrosorption-controlled arrays. Again, this particular system was shown also to operate in harmony with the theory [92]. The study of this effect corroborated the above-described principles, but since it is not electrochemical but rather thermal, we will not dwell on it any further.

**Figure 16:**
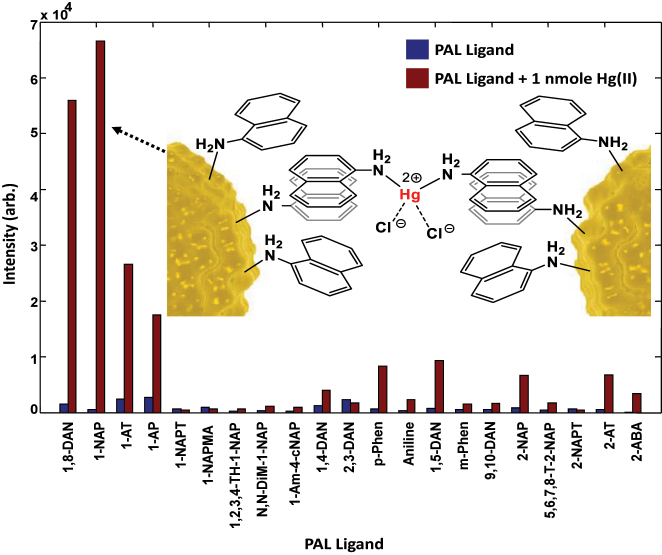
The library of polyaromatic ligands (PALs) spontaneously attaching to AuNPs that form quasi-2-d, flat arrays at a water/DCE interface. Raman signals from PALs as SERS reporters were studied in the presence of Hg ^2+^ ions (brown bars) and in their absence (blue bars) in aqueous solution. A particularly dramatic difference is displayed by 1,8-DAN and 1-NAP pals, the latter considered the best ligand for this mode of detection. Reproduced with permission from Ref. [141]. Copyright 2014 Wiley‐VCH GmbH.

Another principle for formation at LLI (water/DCE) of dense arrays of AuNPs functionalized by polyaromatic ligands (PALs) is based on the gluing effect of heavy ions coming to the interface from the aqueous phase and mediating the linkage between the ligands of the neighbouring NPs [141]. That effect will drive the NPs closer together, thereby intensifying the hot spots between them (Figure 16). The consequence of that phenomenon can be traced to enhancement of the Raman signal from the PALs that themselves also act as the SERS probes. A library of over 20 PALs has been examined in an effort to identify the PALs with the strongest response to mercury ions. The study has shown that e.g. that 1-NAP PAL demonstrates a huge difference in its SERS-signal in the presence and absence of such ions. Such a system can be used as a highly sensitive sensor of the presence of mercury ions in solution: it can detect their quantities down to 10 pmole levels, whilst at the same time differentiating between other heavy metals based on spectral variability.

A similar principle has been used in Ref. [142] for the detection of lead ions, even more important for water quality control. The same idea of bringing AuNPs, now functionalized by glutathione ligands, closer together to form a dense NP array by the bridging effect of Pb^2+^ ions. The effect of tiny amounts of such ions (as low as 1 ion per pair of NPs) has been detected. It was not traced, however, through SERS signals but via optical reflection from the AuNP array at the LLI. As described in this review, the NP array provides a broad reflectance spectrum with a maximum centred in green-yellow part, depending on the size, shape, and material of NPs, and the array density. The reflectance intensity at maximum is substantial (between 20 and 60 % at normal incidence, depending on the size of NPs, 16–40 nm in diameter), which vanishes sharply only when the NPs in the array are separated by more than their diameter. This opens up the use of a wider range of NP’s surface-to-surface separations that provide the effect, as compared against narrow 2–5 nm gaps over which SERS detection is possible. Moreover, unlike Raman or other second order nonlinear optical effects, the simple linear reflectance signal is stronger, requires no signal reporters, and overall needs less sophisticated facilities. The emergence of reflection can be seen with the naked eye: the mirror emerges after the addition of a tiny amount of lead salt.

## 8 Further routes of using chemistry without electrochemistry

The key scenarios of using chemical effects on the photonic system, majorly for detecting gases and chemical reagents have been summarized in Ref [143]. Namely, two main concepts emerged here: (i) in *direct* sensing configurations, the reagents actively influence the plasmonic entity, changing its material properties and thereby its optical response, and (ii) in *indirect* sensing schemes, the plasmonic structure is located adjacent to some reagent-sensitive material and it is affected by and thereby probes the chemically induced changes of the dielectric properties of that material. Progress in this area, also actively using noble-metal NPs, has been overviewed in Ref. [144]. Sensors based on the first scenario are vast, whereas those based on the second one often encountered problems with reversibility.

A characteristic example of the second scheme was recently reported in Ref. [145]. There, a lithography-free, wide-angle, and dynamically reconfigurable subwavelength optical device was constructed, composed of hydrogen sulfide responsive CuO thin film on an optically thick gold (Au) substrate, operating on the principle of the reversible chemical conversion of CuO to copper sulfides (CuS∕Cu_2_S) upon exposure to air containing H_2_S). Switching between two modes of optical responses (high reflectance/high absorption) of the device was observed upon hydrogen sulfide exposure and the corresponding transition from CuO to CuS∕Cu2S. Restoring the CuO was performed by subjecting the sample to 400 C heating. If this is claimed to be the route for reversible reconfiguration, it is not the one that may attract photonics *per se*, although it can be interesting for sensing.

Since this line of works is at best tangential to the main subject of this review, we will not dwell on it any further, having discussed it just to delineate the differences between the “chemical” and “electrochemical” photonics.

## 9 Electrotunability and the speed of response of self-assembling electrochemically controlled optical platforms

The results shown in the previous sections have demonstrated that the physical picture of the optical effects based on controlled electrosorption of NPs has been properly understood, and can be laid into the basis of a variety of electrochemically controlled optical devices. As mentioned, however, making these devices practical requires the miniaturization of electrochemical cells, moving to micro-cells, and micro-compartmentalized cells, to speed up the system response to changing voltage. The response time, when limited by the diffusion of NPs, as at ITIES, is expected, as well as it has been experimentally checked, to be inversely proportionally to the square of the cell thickness [99]. For a 10 μm thick cell, the response time would be in the milli-second domain. Such speed of response would still be too slow for optical communications, but it will be sufficient for many other switchable filter applications. Still, the speed of response will not always be diffusion limited like at an LLI, if there are barriers for NP electrosorption-desorption, as was discovered for solid electrodes. Then, reducing the path for diffusion alone would not solve the problem. One must make sure to remove those barriers. This is generally achieved by broadening the range of potential variation of the electrode. Warning: the latter is limited by the electrochemical potential window, i.e. the diapason of voltages within which no electrochemical reactions of the species of the used electrolytic solution can take place. To overcome this difficulty, one may try some organic electrolytes or ionic liquids, or passivate electrodes. Using pure ionic liquids may be problematic due to their high viscosity which will slow down the diffusion of NPs, but using some small amount of organic additives (creation of “solvent-in-salt” electrolytes) may solve this problem. Although theory may have some navigation effects here, the final answers will be found by experimentation.

## 10 Beyond “simple” systems

All in all, the electronuability of rather standard optical effects, such as absorption, reflection, and transmission of light, in simple systems with standard transparent or reflective optical components involved have been demonstrated. But one can foresee involvement in such systems with more complicated “odd” metamaterial components (of zero [146] or negative refraction index [147, 148]), which would allow us to turn their funky performance on and off. This is a long road. It will take time, effort and money, to go beyond the proof of concept and demonstrate novel practical devices based on those concepts.

Furthermore, electrochemical photonics based on voltage assisted NP assembly may deal with super structures with characteristic dimensions larger than the wavelength of light. A straightforward way to do it is to lay on the surface of an electrode (transparent or reflecting one, subject to the task of interest) an optically and electrochemically inert mask with any structures of interest cut out of the mask, thereby opening those sections to electrosorption of NPs. The control of the population of those sections with NPs by polarizing the electrodes will lead to the variation of the corresponding effects of the optical properties of those sections. The underlying electrode could further be compartmentalized, so you could control the voltage drops and thereby the properties of each section independently. Options here are unlimited, subject to the researchers’ imagination, ideally working in cooperation with cutting-edge gurus from the corresponding branches of photonics. Electro-switchable photonic waveguides/phonic circuits come to mind immediately, to be built resting on the obtained fundamental knowledge.

## 11 Photonics and electrocatalysis

There is one more aspect of relation of photonics and electrochemistry. There were a number of works exploring electrocatalytic properties of self-assembled layers of NPs, such as reported e.g. in Ref. [149–151] (for NP arrays at ITIES). But this field has got a new turn, when combined with the interface illumination. This subject, which has grown already into a new discipline – *reactive plasmonics* [74–76], is very interesting from the fundamental point of view and in principle for applications: e.g. can the formation of NP arrays at electrodes speed up the hydrogen evolution reaction by shining light with plasmon resonance frequency, offering thereby new modes of photo-electrocatalysis, e.g. of the production of hydrogen for hydrogen economy. We will not discuss this here, because many key aspects of this rapidly developing field are not yet well understood. Moreover as intriguing as it is from the fundamental point of view, it is not clear yet how practical such systems could be. Indeed, speaking about liquid/liquid or polished electrode/electrolyte interfaces – those are flat, but not volume-filling, i.e. they have a small area of the interface, unlike those required for industrial applications of electrocatalysis.

Creating, however, decorated novel transparent electrode materials by controlled self-assembly at such interfaces is an interesting alternative [152]. Preparation of solid state nanomaterials using electrodeposition of larger size colloidal particles onto templated electrodes in ordinary electrochemical cells is a separate direction for electrochemical nanotechnology [153].

## 12 Concluding remarks (will “wet photonics” excel?)

Electrochemistry is an amazing interdisciplinary science that had already “donated its blood” to many branches of sciences and engineering, from energy storage to biology and medicine. We have outlined above a new, emerging avenue for it. But would “wet photonics” ever take off? For fundamental investigation – why not, but for practical applications? If it does, the advantages could be enormous ranging from a new class of self-assembling and self-healing structures to fast response filters consisting of tuneable optical properties.

Moving forward, one possible way (not tried and tested yet!) to stabilise these systems is to use room temperature ionic liquids as electrolytes, perhaps slightly doped with an organic solvent to reduce their viscosity but still not undermining their non-volatility. Those behave almost like gels [154, 155], more than the aqueous electrolyte in the Dead Sea, and most of them are optically transparent. The question is, again, whether they could let NPs move easily and fast enough to or away from the electrode, and generally form dense electrosorbed monolayers at the interface? If they do, when implemented in micro-engineered electrochemical cells, they would not be felt wet to a solid-state purist. This must be tried, and not knowing the result in advance, makes it even more exciting!
